# Another cat and mouse game: Deciphering the evolution of the SCGB superfamily and exploring the molecular similarity of major cat allergen Fel d 1 and mouse ABP using computational approaches

**DOI:** 10.1371/journal.pone.0197618

**Published:** 2018-05-17

**Authors:** Rajesh Durairaj, Patrick Pageat, Cécile Bienboire-Frosini

**Affiliations:** 1 Department of Behavioral and Physiological Mechanisms of Adaptation (D-MPCA), Research Institute in Semiochemistry and Applied Ethology (IRSEA), APT, France; 2 Department of Semiochemicals Identification and Analogs Design (D-ISCA), Research Institute in Semiochemistry and Applied Ethology (IRSEA), APT, France; Duke University, UNITED STATES

## Abstract

The mammalian secretoglobin (SCGB) superfamily contains functionally diverse members, among which the major cat allergen Fel d 1 and mouse salivary androgen-binding protein (ABP) display similar subunits. We searched for molecular similarities between Fel d 1 and ABP to examine the possibility that they play similar roles. We aimed to i) cluster the evolutionary relationships of the SCGB superfamily; ii) identify divergence patterns, structural overlap, and protein-protein docking between Fel d 1 and ABP dimers; and iii) explore the residual interaction between ABP dimers and steroid binding in chemical communication using computational approaches. We also report that the evolutionary tree of the SCGB superfamily comprises seven unique palm-like clusters, showing the evolutionary pattern and divergence time tree of Fel d 1 with 28 ABP paralogs. Three ABP subunits (A27, BG27, and BG26) share phylogenetic relationships with Fel d 1 chains. The Fel d 1 and ABP subunits show similarities in terms of sequence conservation, identical motifs and binding site clefts. Topologically equivalent positions were visualized through superimposition of ABP A27:BG27 (AB) and ABP A27:BG26 (AG) dimers on a heterodimeric Fel d 1 model. In docking, Fel d 1-ABP dimers exhibit the maximum surface binding ability of AG compared with that of AB dimers and the several polar interactions between ABP dimers with steroids. Hence, cat Fel d 1 is an ABP-like molecule in which monomeric chains 1 and 2 are the equivalent of the ABPA and ABPBG monomers, respectively. These findings suggest that the biological and molecular function of Fel d 1 is similar to that of ABP in chemical communication, possibly via pheromone and/or steroid binding.

## Introduction

The secretoglobin (SCGB) superfamily comprises small, secreted globular proteins found in mammals [1], such as uteroglobin (UG; also known as club cell secretory protein, Scgb1a1) [2], [3], UG-like proteins (Scgb3a family) [4], mammaglobin (MG; Scgb2a2) [5], [6], mouse androgen-binding protein (ABP; Scgb1b and Scgb2b families) [7], [8], Fel d 1 [9], [10], and prostate steroid-binding protein (PBP; Scgb1d and Scgb2a families) [11]. Although the functions of nearly all of these proteins are unknown functions, UGs are multifunctional proteins with anti-inflammatory/immunomodulation properties [12]. Club cell secretory protein was the original UG/blastokinin, which was renamed Clara cell secretory protein and then ultimately renamed again as club cell secretory protein because of the World War II shadow cast over Max Clara [13]. These proteins and their various functions are classified within the same family [1] based on two common traits: i) SCGB members are small globular proteins with a conserved cysteine-rich domain and subunits that consist of four α-helix bundles in a boomerang configuration (the secretoglobin fold), creating a hydrophobic binding pocket [14]; and ii) these proteins are secreted in many tissues, such as the uterus [15], prostate [11], lungs [4], and sebaceous glands [16], [17], [18], and other glands of the face and neck [7], [19], [20].

Among the SCGB members, Fel d 1 is the major allergen (Mall) produced by cats (*Felis catus*), eliciting the production of specific immunoglobulin E (IgE) in 85% to 95% of allergic patients and causing respiratory diseases such as asthma or allergic rhinitis/rhinoconjunctivitis [21], [22]. This allergen is a 35 kDa tetrameric glycoprotein composed of two heterodimers (subunits A and B) with a dimerization interface [10], [23]. Each heterodimer consists of two polypeptide chains linked by three disulfide bridges: chain 1 contains 70 residues; chain 2 contains 90 or 92 residues; and the two chains are encoded by independent genes [24], [25]. Chain 2 contains an N-linked oligosaccharide composed of triantennary glycans [26]. Variants of Fel d 1 have been described for many years, and their impact on allergenicity has already been demonstrated [27], [28].

Fel d 1 is abundantly released into the environment by cats [29], [30]. The primary anatomical sites of Fel d 1 production are the facial area and anal sacs [17], [18], [31]. Interestingly, these areas are also involved in intraspecific chemical communication in cats through the release of pheromones [32], and Carayol et al. (2000) hypothesized that Fel d 1 production at various anatomical sites are also involved in the binding of pheromones [18]. The existence of structural homologies between Fel d 1 and steroid-binding proteins [33] may suggest that Fel d 1 plays a role in sexual communication through the binding of specific ligands. The immunological properties of Fel d 1 have been linked to cat sex and behavior [34]. However, the specific biological function of Fel d 1 remains unidentified, despite several hypotheses about its role in feline chemical communication [18], [34].

Several studies have shown that the sequence and structural alignment of Fel d 1 are 50% identical to ABPα [33], [35] with chain 1 and chain 2 of Fel d 1 being closely related to ABPα and ABPβγ, respectively [36]. Secreted ABP and Fel d 1 are both applied to animal pelts [16], [29], [37]. The Karn/Laukaitis research team has published several articles and reviews describing ABP gene nomenclature [36], and the chromosomal location of salivary ABP [38], [39]. They have also evaluated the evolutionary relationships of salivary ABP subunits [40] and ligand binding by the dimer [41]. An evolutionary bloom of ABP clades has been identified within the mouse SCGB gene superfamily [42] leading to a claim of orthology between human and mouse *Abp* genes. However, these phylogenetic data are questionable in light of more recent findings that the sequences used by Jackson et al [42] in human/mouse phylogenies are partial introns and retrotransposons [43].

Mouse salivary ABP is considered to be a proteinaceous pheromone that mediates assortative mate selection. The three ABP subunits (ABPA27, ABPBG27, and ABPBG26) form A27:BG27 (AB) and A27:BG26 (AG) dimers in the saliva’s of both sexes, and experiments using an *Abpa27-Abpbg27* knockout strain have shown that these genes are required for ABP-based recognition [44]. Assortative mate choice based on this subspecies recognition mechanism leads to reinforcement in the European mouse hybrid zone where the two subspecies meet [45], [46]. Additionally, ABP is capable of binding steroids deposited on the animal’s pelt during grooming and may play a role in ABP-based scent marking detected by females during mate selection [37].

In earlier experimental studies, the mouse genome was shown to contain 30 *Abpa* and 34 *Abpbg* subunit genes in a 3 Mb region on the proximal end of mouse chromosome 7 [47]. *Abpa* and *Abpbg*-like genes were further mapped to the tertiary structure of Fel d 1 using positive site selection [48] suggesting that that their shared evolutionary history may have produced similar molecular and, possibly functional, features between Fel d 1 and ABP. Therefore, we combined several kind of computational methods (that attempt to extract functional information from protein sequence and structural data) to provide insightful cues for the identification of molecular similarity between Fel d 1 and ABP, since their molecular and structural similarities could provide evidences of similar biological functions. Hence, the present study focuses on i) the evolutionary clustering pattern of the whole SCGB superfamily and the relationship of Fel d 1 chains with paralogs of ABP in particular; ii) comparison of conserved and functional binding sites between Fel d 1 and ABP; and iii) predictions based on structural modeling, superimposition, and simulations as well as Fel d 1 and ABP dimer-steroid docking. Therefore, this investigation aimed to reveal the putative biological and molecular functions of Fel d 1 through comparison of the sequence and structural annotations of ABP subunits from a chemical communication viewpoint.

## Methods

### Computational features

**Fig 1:**
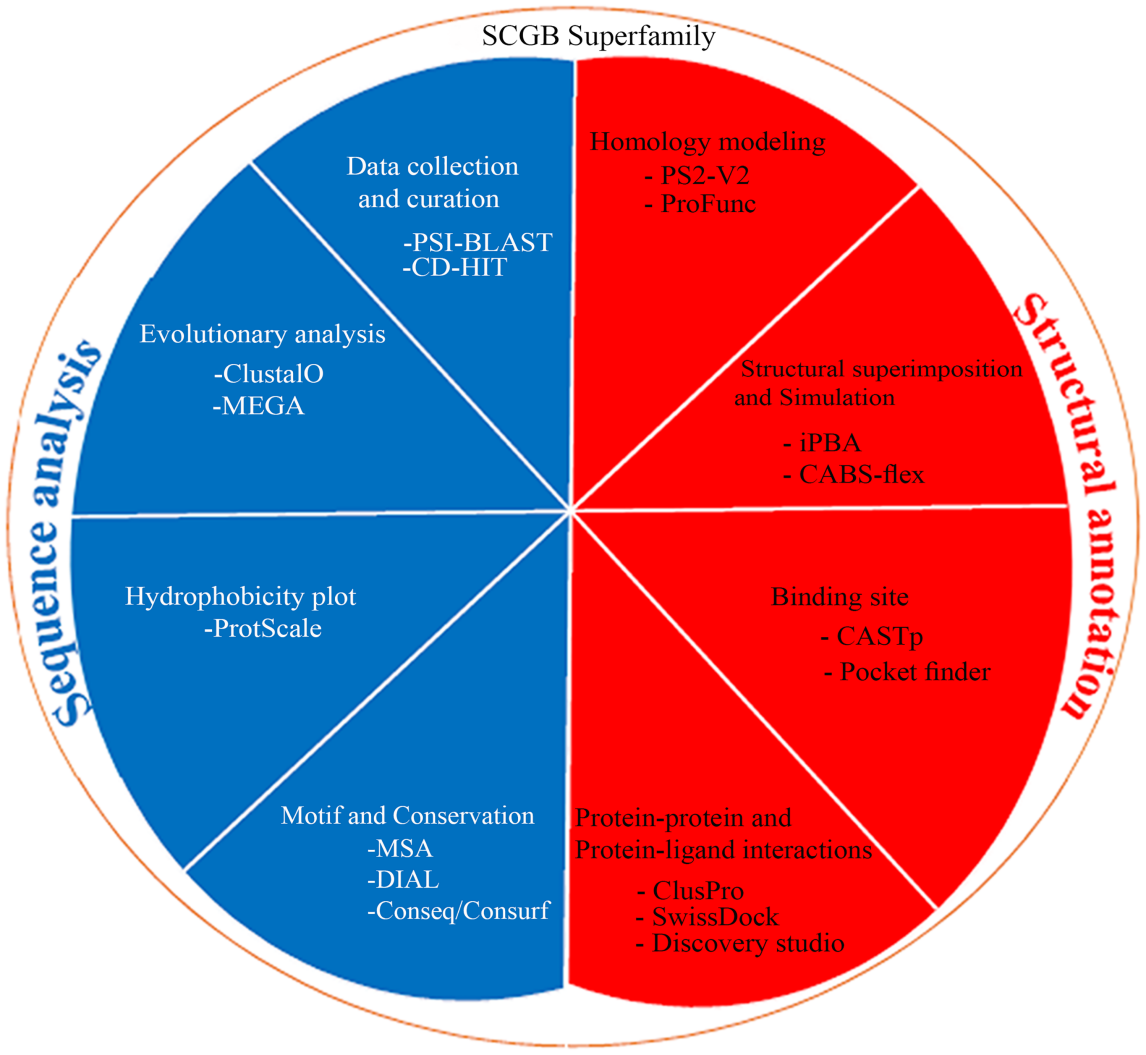
Computational methodology. Graphical representation of the sequence and structural annotation methods were employed for the SCGB dataset.

In this study, SCGB sequence analysis and structural annotation were graphically represented (Fig 1). Additionally, computational analyses were employed throughout the study to investigate the molecular similarities between Fel d 1 and ABP. All analyses were carried out in a high-performance computer with various computational tools and servers.

### Collection and curation of SCGB sequences

Protein sequences (~1950) from the whole SCGB superfamily were obtained from the National Center for Biological Information (NCBI) (http://www.ncbi.nlm.nih.gov/) and UniProt (http://www.uniprot.org/) databases. All uncharacterized, hypothetical, unknown, third party annotation (TPA), patented, and synthetic construct entries were removed from the datasets. Subsequently, all SCGB proteins (~1080) were selected and evaluated with the position-specific iterative basic local alignment search tool (PSI-BLAST) to produce aligned proteins with standard *E*-value thresholds [49]. The preliminary dataset (in FASTA format) was processed with the CD-HIT web server (http://cd-hit.org/), which was used to remove redundant sequences showing ≥90% identity [50]. Then, the nonredundant SCGB dataset was combined with UniProt reviewed entries to perform CD-HIT analysis in a curated dataset, with an identity cut-off of 0.9.

#### Screening of the curated dataset

**Fig 2:**
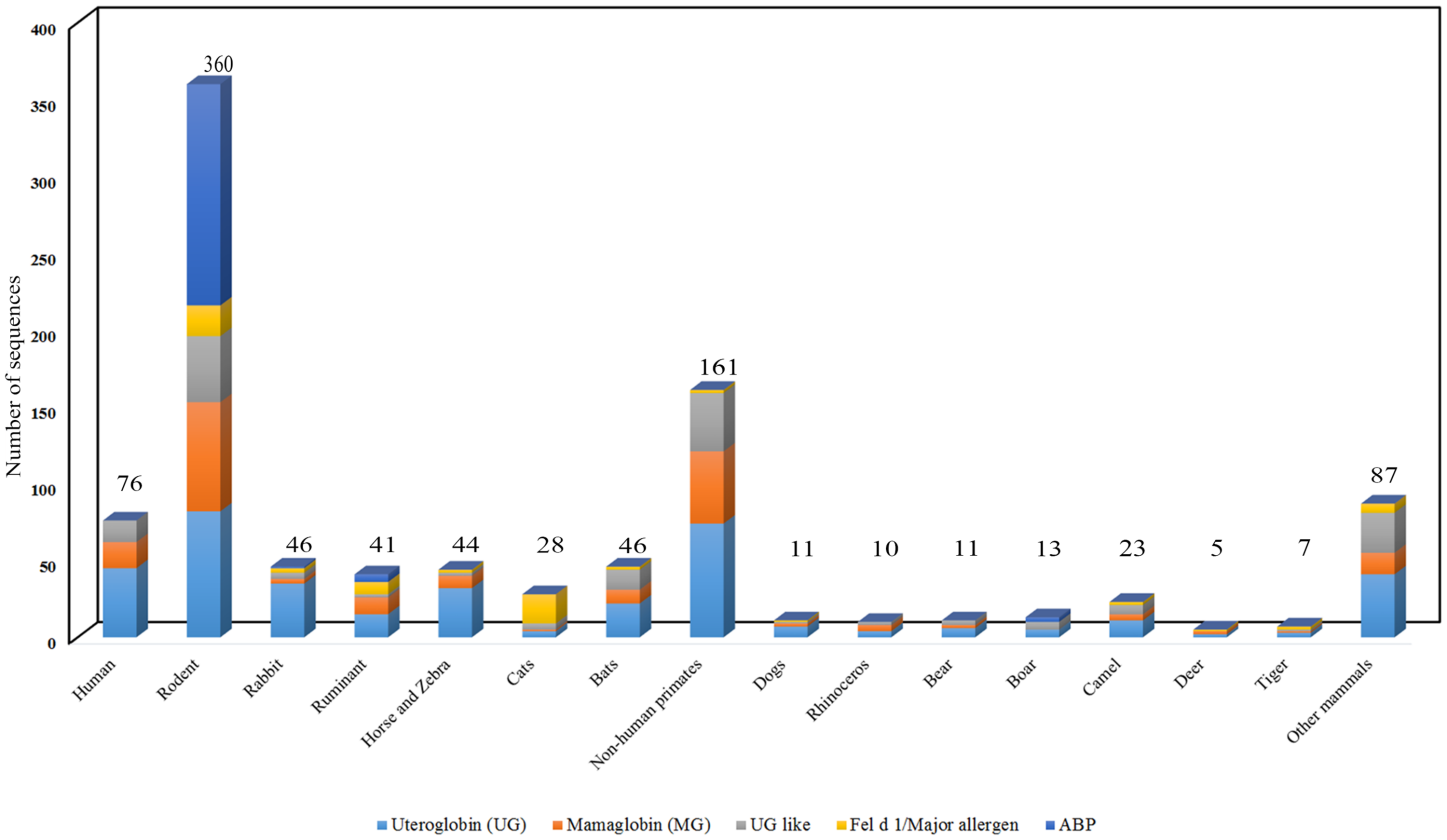
Dataset collection. All sequence in the SCGB protein dataset (969) sequences were separated by subtypes and classified by organism. Rodents and non-human primates exhibit more SCGBs than other mammals. The numbers of Fel d 1 chains and ABP subunit sequences are greater in cats and rodents, respectively, compared with those in other mammals. UG and related sequences are present in all species.

Nonredundant SCGB sequences (969) were obtained and classified by organism with subtypes (Fig 2). The cumulative SCGB protein family members (126), Fel d 1/Mall (20) and ABP (47) were retained and renamed with the short organism names of the SCGB protein identifiers for further computational analysis (S1 File). The geographical origin of species and their short zoological names were tabulated (S1 Table). Additionally, a list of 28 *Abp* paralogs without pseudogenes was obtained from an earlier experimental report [8] and tabulated along with protein IDs (S2 Table) and the 64 *Abp* paralogs with pseudogenes (S2 File).

### Construction of an evolutionary tree

#### Multiple sequence alignment

Curated SCGB proteins and Fel d 1/Mall-ABP were submitted to multiple sequence alignment (MSA) using the ClustalO program (http://www.ebi.ac.uk/Tools/msa/clustalo) with default parameters. Many unaligned indels and gappy regions were edited manually by reviewing the ClustalW alignment file (.aln) in Molecular Evolutionary Genetics Analysis (MEGA) V.6.0 software [51]. MEGA software enables the calculation of aligned residue site p-distances of the proteins and provides a graphical user interface (GUI) to support precise sequence alignment [52]. The final sequence alignment session (.mas) was exported in MEGA (.meg) file format to generate a clustering pattern for the SCGB superfamily proteins.

#### Phylogenetic methods and divergence time tree

The sequence alignments of the SCGB, Fel d 1/Mall-ABP, and Fel d 1-ABP paralog protein datasets were employed to construct a phylogenetic tree, adopting maximum likelihood (ML) and the best substitution Jones-Taylor-Thornton (JTT)+G+I model [53]. MEGA was used to determine the divergence pattern and branch length between Fel d 1/Mall-ABP in various species. The accuracy of the phylogenetic distribution of branches was determined based on 1000 bootstrap (BS) replicates, and gappy regions/extra indels were treated as complete deletions via the ML heuristic phylogenetic inference method. The percentage identity matrix (PIM) values of the Fel d 1/Mall-ABP sequence alignment scores were calculated from the best match (67 X 67 substitution) similarity and differences in the default BLOSUM62 matrices [54]. Exact sequence identity and nonexact identity were estimated from pairwise distance alignment, which is a sensitive method for detecting distant relationships. Additionally, the evolutionary pattern and divergence time tree of Fel d 1 and 28 mouse ABP paralogs were calculated using the RelTime-ML method in MEGA Time Tree Wizard. The divergence time tree was inferred via the ML method based on the JTT matrix model, and the time tree showed the highest log likelihood (-1859.1085) of the Fel d 1-ABP paralogs.

### Hydrophobicity plot

The Fel d 1 (PDB ID: 2EJN) and ABP paralogs (ABPA27, ABPBG27, and ABPBG26) were employed for the construction of hydrophobicity plots (window size = 9) using a Kyte-Doolittle method [55] with the ProtScale (http://web.expasy.org/protscale) tool from the ExPASy bioinformatics resource portal.

### Functional sites and conservation prediction

The Fel d 1-ABP paralogs were employed for the prediction of functional sites and domains in proteins using the DIAL webserver (http://caps.ncbs.res.in/DIAL/DIALserver.html). The motif, UG signature, and identical and conserved residues of Fel d 1 were predicted based on sequence alignment with ABP subunits and dimer sequences (A27:BG27 (AB); A27:BG26 (AG)). The applied ABP nomenclature follows earlier reports [36], [38]. Specifically, the sequences of other proteins involved in chemical communication (the secretoglobin pheromaxein from pig and cat protein Fel d 4 from the lipocalin family) were compared with the Fel d 1 sequence to predict similar functional motifs and identical ligand-binding residues hypothetically linked to chemical communication functions. ABP subunits were employed to predict residue conservation and solvent-accessible surface buried/exposed residues using the ConSurf server (http://consurf.tau.ac.il) [56]. The conservation score was predicted via a Bayesian method with a JTT evolutionary substitution model.

### Homology modeling and validation of ABP models

ABPA27, ABPBG27, ABPBG26, AB, AG and 5 other selected subunits of ABPA (of NCBI ID: AGJ84407.1, NP_001257472.1, AAM08256.1, AAB97170.1, AAM08258.1) were employed to construct a three-dimensional model using the (PS)^2^-V2 (http://ps2.life.nctu.edu.tw) [57] automatic homology modeling server. Then, the homology models were validated using Ramachandran plots constructed on the ProFunc server (http://www.ebi.ac.uk/thornton-srv/databases/profunc) [58], in which topology and residue interactions were observed. Further analyses were applied to the 10 validated homology models that included AB and AG dimers.

### Superimposition and simulation of ABP models

The selected ABP models with the template (PDB ID: 2EJN) were 3D superimposed, and a predicted structural alignment comparison was conducted on the iPBA webserver (http://www.dsimb.inserm.fr/dsimb_tools/ipba/example.php) [59]. Root mean square deviation (RMSD) was calculated from a pairwise comparison of local backbone conformations and dihedral angles in the corresponding models with the template. The ABP model simulation were carried out via the CABS-flex pipeline (http://biocomp.chem.uw.edu.pl/CABSflex/index.php) [60] server to predict the structural flexibility of folding patterns and protein conformational changes. Protein conformations provide a better understanding of structural arrangements, which have an impact on stability, drug design and protein functions and interactions as well as protein engineering analyses [59]. The residual fluctuation of Cα atoms was computed using the backbone building from quadrilaterals (BBQ) algorithm and the ModRefiner method. The simulation outputs were equal to 10 ns (nanoseconds) for near-native dynamics. The propensity of the residue mean-square-fluctuations (RMSF) profiles was collected for each selected ABP model.

### Binding site analysis

The structurally validated ABP models were submitted to the online CASTp (http://sts.bioe.uic.edu/castp) server to predict ligand-binding sites. Binding cavities were characterized via the solvent accessible surface (SA, Richards’ surface) and molecular surface (MS, Connolly’s surface) methods. The alpha shape theory was used to determine the area-volume of protein pockets and cavities.

### Fel d 1-ABP and ABP-steroid docking

The protein-protein interaction tool was used to highlight the structural resemblance and surface binding site contacts between Fel d 1 and ABP dimers. There is presently no evidence that these proteins are involved any physical contact in natural context. Hence, we artificially simulated their binding ability to identify the surface binding contacts and residue similarity of the two proteins. The AB and AG dimers and 5 other ABPA models were included for molecular docking with heterodimeric subunit A of Fel d l. Molecular docking of Fel d 1-ABP was performed with the ClusPro algorithm (https://cluspro.bu.edu) using fast Fourier transform (FFT) programs. ClusPro is an automated docking server for the prediction of protein surface binding [61]. Similarly, the AB and AG dimers were used for the analysis of steroid interactions at the Swiss-Dock server [62]. Steroid molecular structures, such as those of progesterone (P), testosterone (T), and dihydrotestosterone (DHT), were collected from PubChem (https://pubchem.ncbi.nlm.nih.gov/), and these structures (.sdf) were converted into the desired (.mol2) format using Open Babel software. The best-interacting residues were predicted with Discovery studio V.2.1 (Accelrys, San Diego, USA) (http://accelrys.com/products/discovery-studio), and the binding models were visualized with UCSF Chimera using the ViewDock plugin [63].

### Data archiving

The ten best validated 3D coordinate ABP models were deposited in the figshare database (https://figshare.com). The protein models are available in the database (doi:10.6084/m9.figshare.6236537.v2).

## Results

In the present study, we performed evolutionary clustering of SCGB superfamily proteins and predicted the molecular similarities of Fel d 1 with ABP paralogs through various computational analyses. The boomerang-shaped resemblance and identical functional sites of ABP led us to investigate whether similar molecular and biological functions were present in Fel d 1. Here, our primary hypothesis was to determine similar molecular features, surface structural clefts, and protein-protein docking between Fel d 1 and ABP dimers. Indeed, we revealed the close evolutionary relationship between Fel d 1 and ABP via clustering in a phylogeny of the SCGB superfamily of proteins. Additionally, we modeled the steroid binding of AB and AG dimers and showed that ABP dimer-steroids interacting sites were correlated with the binding sites of Fel d 1, consistent with Callebaut et al [14]. Finally, computational tools were used to construct ABP structural models, with predictions of similar structural features between Fel d 1 and ABP dimers.

### Clustering pattern of SCGB superfamily proteins

**Fig 3:**
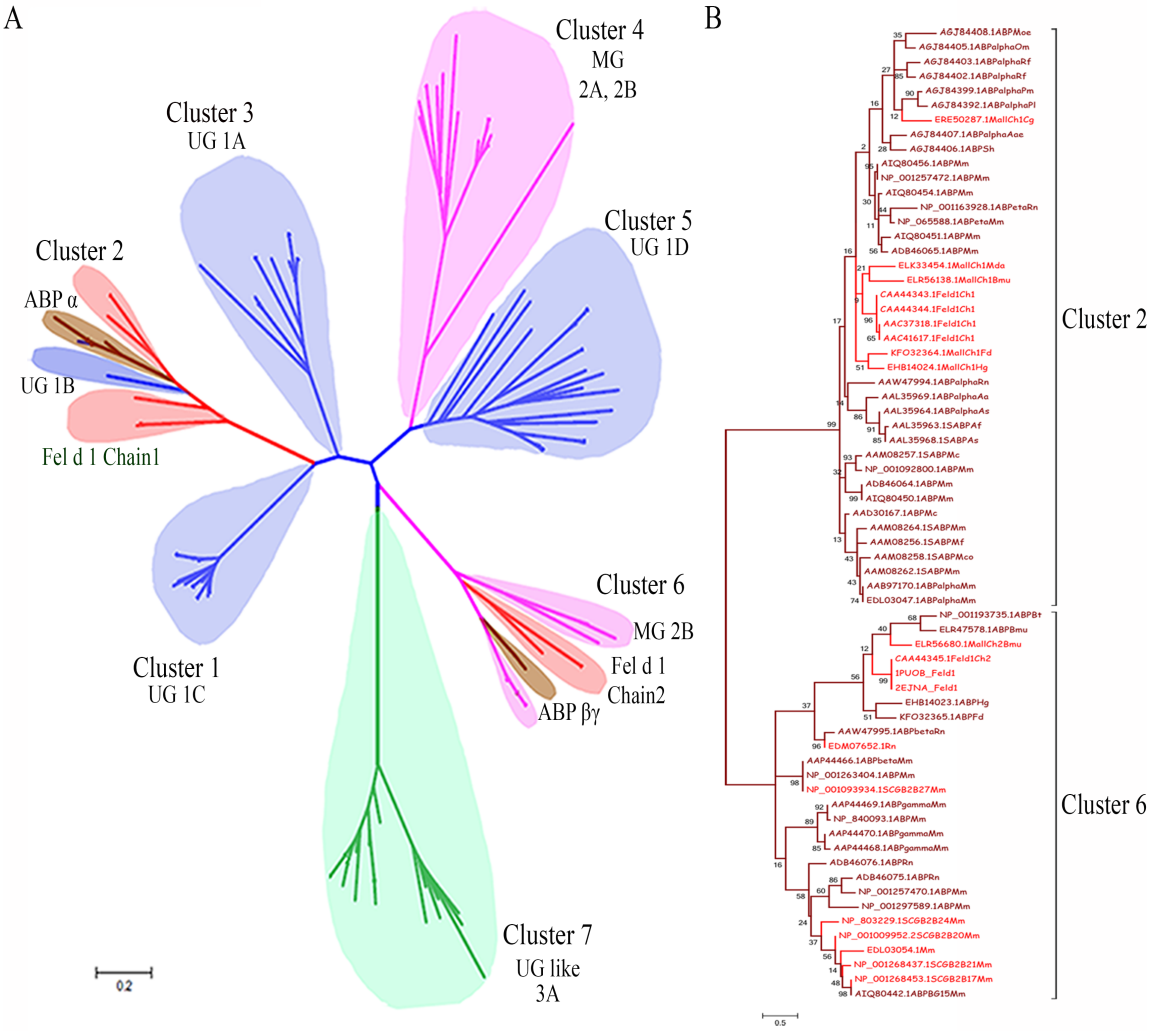
Construction of the phylogenetic tree and clustering branching pattern of Fel d 1 and ABP. (A). The radial evolutionary tree was generated using selective members of the SCGB superfamily proteins from different organisms. The UG, MG, UG like, Fel d 1/Mall, and ABP are represented as blue, pink, green, red, and brown, respectively, using a subtree coloring scheme, which provides the distribution of distantly related SCGB superfamily members. The SCGB divergence tree was estimated using a scale bar of 0.2 amino acid substitution matrices per site. (B). Phylogenetic tree of clusters 2 and 6. The dendrogram of Fel d 1/Mall-ABP showed clustering pattern of chains 1 and 2 of Fel d 1. Fel d 1/Mall-ABP was estimated using a scale bar of 0.5 amino acid substitution matrices per site and the BS values indicated on the tree branches.

The selected SCGB, Fel d 1/Mall, and ABP datasets were used to construct a phylogenetic tree (Fig 3A). The radial tree view showed palm-like clustering with unique protein clades. The tree was distributed across seven major clusters, and the UG proteins were reliable members of all subtypes of SCGB proteins. The evolutionary tree showed UG, MG, and UG-like proteins in distinct clusters.

#### Clusters 1/3 and 5

UG protein members 1C, 1A, and 1D were exclusively distributed in clusters 1, 3 and 5, respectively (blue). Additionally, three UG cluster 1, 3, and 5 genes were obtained from the NCBI and UniProt databases (S3 File). Each of the UG clusters contained unique protein (1A, 1B, 1C, 1D) members, and the 3^rd^ and 5^th^ UG clusters emerged between the other clusters. In particular, type 1D UG proteins were associated with pig (*Sus scrofa*) pheromaxein in the 5^th^ UG cluster (S3 File).

#### Clusters 2/6, 4/6 and 7

Chain 1 of the Fel d 1/Mall-ABPA subunit and chain 2 of the Fel d 1/Mall-ABPBG subunit were coordinated as neighbor members in clusters 2 and 6, respectively. The divergence of clusters 2 and 6 was directly inverse and associated with UG (blue) and MG (pink) members. Cluster 4 included a separate clade for MG proteins (type 2A and 2B). In cluster 6, ABP (brown) and Fel d 1 (red) were combined with MG 2B proteins as neighbor members. The 7^th^ cluster consisted of a unique dataset of UG-resembling type 3A proteins (green) arranged as cocluster proteins in various organisms. The sequence was aligned appropriately in a single cluster with two subclades. The radial view of SCGB superfamily proteins shows a clustering branching pattern (Fig 3A). Likewise, protein members were included in the curve and circular phylogeny (Panels A and B in S1 Fig).

### Dendrogram of Fel d 1/Mall and ABP

The dendrogram of Fel d 1/Mall-ABP was used to observe the evolutionary distribution of two distinct clusters (2 and 6), and different ABP subunits were included to reveal the clustering pattern with chains of Fel d 1. In cluster 2, chain 1 of Fel d 1/Mall coclustered with ABPA27, and the other mouse ABPA proteins were dispersed within this clade. In cluster 6, chain 2 of Fel d 1/Mall coclustered with ABPBG27 and ABPBG26, and the other ABPBG proteins were dispersed within this clade (Fig 3B). Divergence distance was used to calculate the branch length, radial view of deviation, and circular unrooted tree of Fel d 1/Mall-ABP (Panels A-C in S2 Fig). Furthermore, sequence divergence was analyzed with PIM (S3 Fig), where clusters 2 and 6 of PIM showed the greatest deviation of sequence divergence (0–30%). Additionally, cluster 6 displayed more variability of identity than cluster 2, with a 30% lower percentage of identity being observed in cluster 6 than that in cluster 2. The coclusters of Fel d 1 showed maximum sequence identity with ABP subunits within each cluster, consistent with earlier observations [40]. The results revealed that chains of the Fel d 1 sequence were distributed across completely different subunits of ABP in 23 organisms, and these findings suggest that evolutionary changes occurred in ancestral lineages.

### Evolutionary pattern and the divergence time tree of ABP paralogs

**Fig 4:**
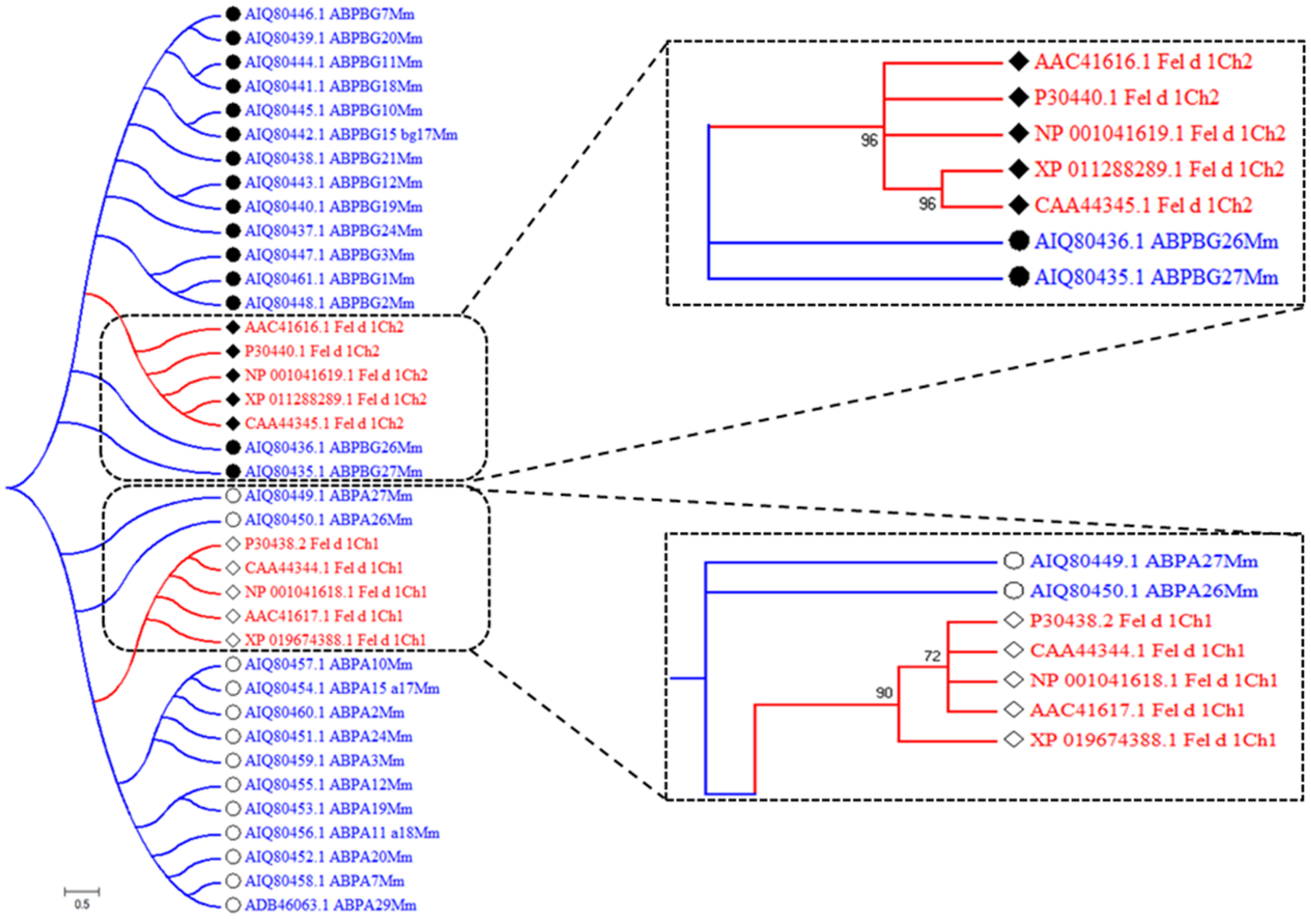
Evolutionary pattern of Fel d 1 and ABP subunits. The phylogenetic tree shows the evolutionary pattern of Fel d 1 and 28 paralogs. Chains 1 and 2 of Fel d 1 cluster are enlarged, and the arrangement of coclusters with ABP paralogs is shown. Chain 1 of Fel d 1 or ABPA is depicted as an open diamond or open circle, respectively. Similarly, the chain 2 of Fel d 1 or ABPBG is depicted as a closed diamond or closed circle respectively. The Fel d 1-ABP paralogs were estimated using a scale bar of 0.5 amino acid substitution matrices per site.

An evolutionary tree was constructed using Fel d 1 and 28 mouse ABP paralogs without pseudogene sequences. Interestingly, the results showed that chain 1 of Fel d 1 coclustered with ABPA27 and ABPA26, and chain 2 of Fel d 1 coclustered with ABPBG26 and ABPBG27 members (Fig 4). Additionally, the ABP paralog dataset was used to build a divergence time tree of Fel d 1- ABP paralogs, and the tree was estimated with lineage and calibration constraints (S4 Fig). The curve time tree was generated between 0.0 to 2.5 million years ago (MYA). The distribution of ABP paralogs emerged with low divergence, showing similarity to Fel d 1. Specifically, ABP subunits such as AIQ80449.1_ABPA27Mm, AIQ80435.1_ABPBG27Mm, and AIQ80436.1_ABPBG26Mm originated from the ancestral node of Fel d 1-ABP proteins. Here, the ABP subunits are the most likely ABP orthologs of chains 1 and 2 of Fel d 1. Notably, compared with other ABP subunits, the ABPA27 subunit shared 50% sequence identity with Fel d 1 (see also [33]), and the time tree scale was employed to estimate the divergence time.

### Hydrophobicity plot

The combined hydrophobicity plot revealed that the hydrophobic residue values of chain 1 of Fel d 1 were more similar to ABPA27 than other ABP subunits (Panel A in S5 Fig). The residues were aligned to identify the highest probability of residue matches with ABPA. The significant residue pattern of Fel d 1-ABPA displayed an equivalent functional consensus with hydrophobic sites. A lower hydrophobic residue peak was observed at the protein dimer interface in Fel d 1 subunit A (PDB ID: 2ejn_A) (Panel B in S5 Fig).

### Functional site and conservation prediction

**Fig 5:**
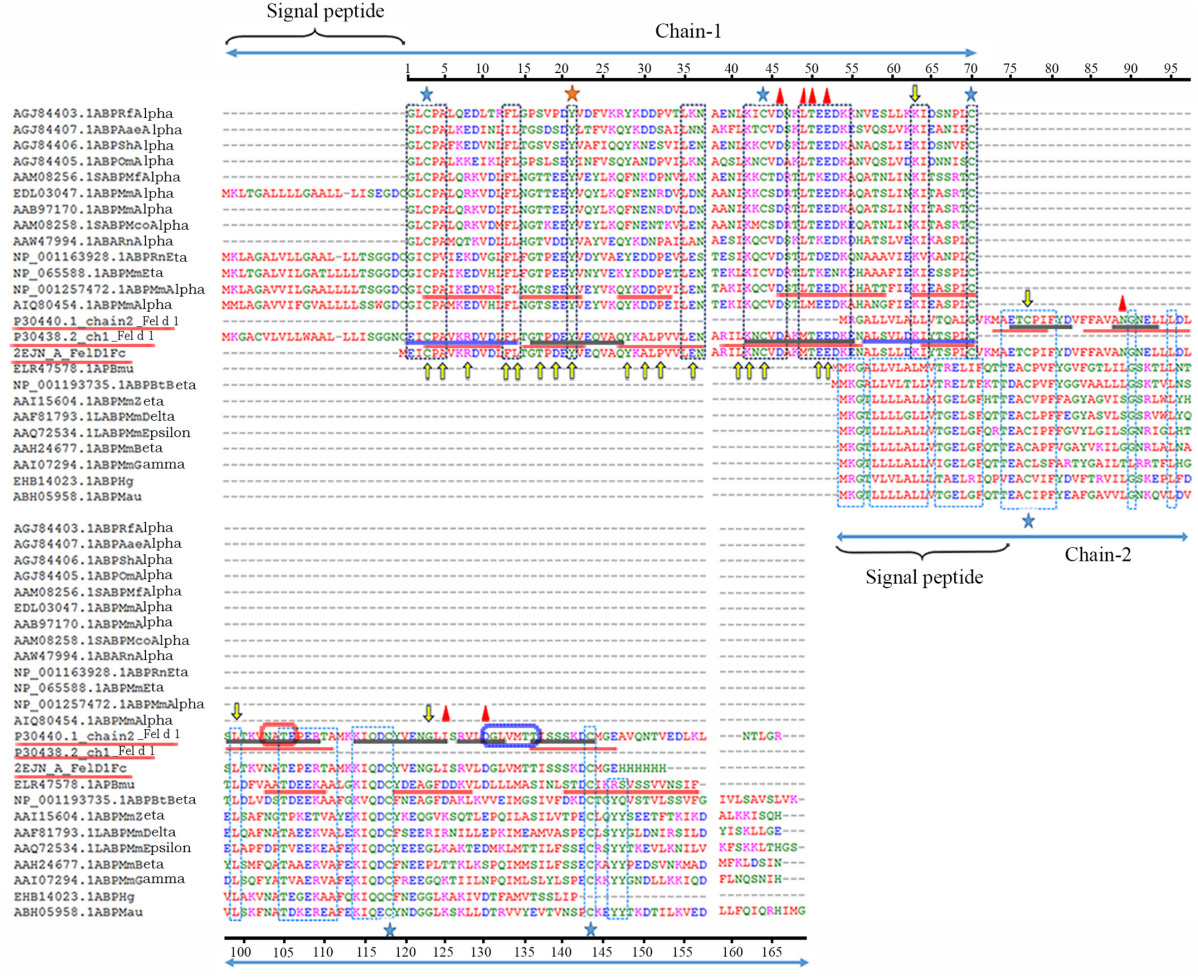
Illustration of sequence similarity and putative sites of Fel d 1 and ABP using MSA. Multiple functional site information is described in the MSA with a residue scale. The Fel d 1 chains 1 and 2 sequences were aligned with various subunits of ABP sequences in MSA. In general, identical and conserved residues are indicated with differently colored dotted boxes: black for chain 1 and blue for chain 2. The cysteine-rich residues are indicated with blue stars, and the unique conserved residue, Tyr21, is denoted with an orange star. The calcium-binding sites are identified with red triangles in subunits A and B of Fel d 1. UG signature and motif sites are indicated with blue, red, and black underlining, respectively. The similarity of the functional sites from pheromaxein is indicated with yellow arrows. N-glycosylation (-NATE-) and N-myristylation sites in chain 2 are indicated with red and dark blue boxes, respectively.

Fel d 1 has three functional domains: a UG family signature 2 domain, a casein kinase II phosphorylation site and an N-glycosylation site (-NATE-). Similarly, ABP contains a UG family signature 2 domain, an N-glycosylation site and a casein kinase II phosphorylation site. Moreover, ABP motif sites were predicted and were found to be identical to Fel d 1 motif sites in chain 1 (-CPA-, -FL-, -EYV-, -LXN-, -KXCVD-, -TEEDK-, -KI-, and -LC-) and chain 2 (-EXCXXF-, -NGN-, -LLXXL-, TEXEX-, -KIQDC-, and -DCM-) (Fig 5). Calcium binding residues Asp46, Met49, Glu52, Asn89, Ile125, and Asp130 are indicated in Fig 5. ABPA27 showed a sequence identity of 51.43% to chain 1 of Fel d 1. Furthermore, ABPBG27, and ABPBG26 shared sequence identities of 34.33%, and 31.34%, respectively, with chain2 of Fel d 1. Similarly, the AB and AG dimers showed identities of 43.07% and 40.88%, respectively, with heterodimeric Fel d 1 subunit A. Identical motif sites are denoted with (*) in the sequence alignment (Panels A-D in S6 Fig). We further investigated putative functions in chemical communication by confirming that chains 1 and 2 of Fel d 1 shares identical residues with pheromaxein C (26.69%), pheromaxein A (24.04%), respectively, which are the subunits of a pig/hog protein capable of binding steroid pheromone [64]. The heterodimeric Fel d 1 subunit shares identical residues with Fel d 4 (27.27%), a cat protein from the lipocalin superfamily described as a kairomone [65]. Moreover, identical prominent residue motifs were present in Fel d 1 compared with pheromaxein and Fel d 4 (Panels A-C in S7 Fig). We predicted 10 functionally exposed and 2 structurally buried residues in chain 1 of Fel d 1 that were highly similar to the conserved sites of ABPA. Six functionally exposed and 1 structurally buried residues of chain 2 of Fel d 1 corresponded to residues in the ABPBG subunits, which were predicted using a neural network algorithm-based method on the ConSurf server. In general, the structurally buried residues Tyr21, Ala38, and Cys42 were identified in Fel d 1 and mouse ABP, which is highlighted in Table 1.

**Table 1:** Prediction of functionally exposed and structurally buried conservation sites in chain 1 and 2 of Fel d 1 with ABP subunits (ABPA-alpha; ABPBG-beta/gamma).

Conservation sites in chain 1 of Fel d 1		Conservation sites in ABPA		Conservation sites in chain 2 of Fel d 1		Conservation sites in ABPBG	
Functionally Exposed	Structurally buried	Functionally exposed	Structurally buried	Functionally exposed	Structurally buried	Functionally exposed	Structurally buried
**Cys3**	**Tyr21**	**Gly1**	**Tyr21**	**Thr29**	Phe4	Cys1	**Cys42**
**Asn37**	**Ala38**	**Cys3**	**Ala38**	**Glu32**	Leu19	Phe4	
**Lys42**		**Ala5**	Cys44	**Lys38**	Leu23	**Thr29**	
**Asp46**		**Asn37**	Leu49	**Gln40**	Ala35	**Glu32**	
**Thr50**		**Lys42**		**Glu45**	**Cys42**	Glu37	
**Glu52**		**Asp46**		**Arg51**	Cys67	**Lys38**	
**Asp53**		**Thr50**		**Ser64**		**Gln40**	
**Lys54**		**Asp53**		**Arg86**		**Glu45**	
**Lys63**		**Lys54**				**Ser64**	
**Cys70**		**Lys63**				Glu66	
		**Cys70**				Cys67	

The bold type residues of Fel d 1 were highly mapped to ABP subunits.

### Homology modeling and superimposition

**Fig 6:**
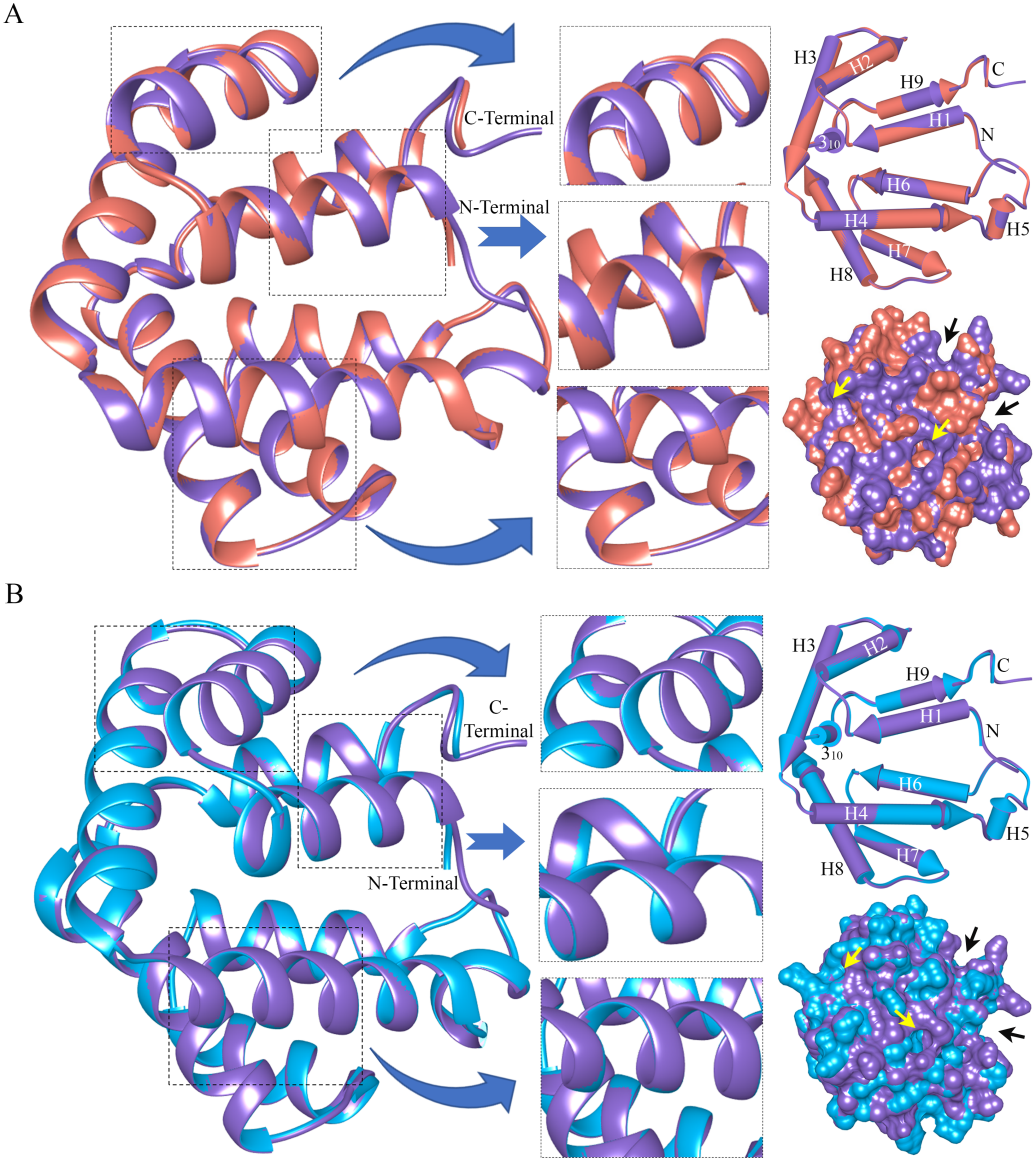
Structural superimposition of subunit A of Fel d 1 with AB and AG dimers. (A) Overlap of the AB (red) structural model with Fel d 1 (purple). (B) Overlap of the AG (light blue) structural model with Fel d 1 (purple). The pipes and plank and molecular surface views represent good superimposition of both proteins. Projections of the superimposition and topologically equivalent positions are shown in dotted boxes. The black and yellow arrows denote the surface cavity in the superimposed structures.

The determination of structural alignment is essential to produce predictive models and protein folding patterns. The selected and validated homology model of ABP paralogs (ABPA27, ABPBG27, ABPBG26, and AB and AG dimers) was created using the best hit template (PDB ID: 2EJN_A). The ABP subunits and dimers were validated with >90% of the most-favored regions (MFRs) from Ramachandran plot scores. The ABP models exhibit similar continuous alpha helices with interconnecting loops, and the conserved residues are indicated as molecular sticks in the structures (Panels A-E in S8 Fig). The heterodimeric Subunit A of Fel d 1 (template) was also structurally superimposed and aligned with the AB and AG dimers. The results showed that the lowest RMSD with the highest global distance test total score (GDT_TS) values supported the folding conformation. Overlap of the AB and AG dimers showed three different topologically equivalent positions, and the pipes and plank in a surface view of the dimers are illustrated (Fig 6). The Ramachandran plot and superimposition GDT_TS scores of the validated ABP models are tabulated (Table 2).

**Table 2:** The selected and validated homology model of ABP with structural superimposition to Fel d 1.

S. No	ABP accession ID	Template	Structure Aligned (Ps2-v2) (%)	SS identity (%)	Ramachandran Plot (Homology Model validation)	GDT_TS (%)
1	ABPA27	2ejn_A	76.09	90	98.5%-MFR 1.5%-AAR	46.76
2	ABPBG27	2ejn_A	75.82	85.51	93.2%-MFR 6.8%-AAR	41.08
3	ABPBG26	2ejn_A	75.79	81.94	90.0%-MFR 10.0%-AAR	43.89
4	AB	2ejn_A	86.06	87.32	92.2%-MFR 6.2%-AAR 1.6%-GAR	96.94
5	AG	2ejn_A	86.06	87.32	91.4%-MFR 7.8%-AAR 0.8%-DAR	96.94

MFR, Most favored regions; AAR, Additional allowed regions, GAR, Generously allowed regions, DAR, Disallowed regions; GDT_TS, Global distance test total score. The GDT_TS provides quality of superimposition with the template.

Dynamic simulations of the ABPA27, ABPBG27, and ABPBG26 models were employed for energy minimization and for the calculation of RMSF. The structural flexibility of ABPA showed a fluctuation plot, similar to what was found for Fel d 1. The lowest RMSD and maximum cluster density revealed that ABPA presented a similar Cα atom local backbone conformation to and shared structural folding with Fel d 1. Dynamic simulations of the structural deviation and fluctuations of ABP subunit residues are presented (Panels A and B in S9 Fig).

### Binding site prediction

**Fig 7:**
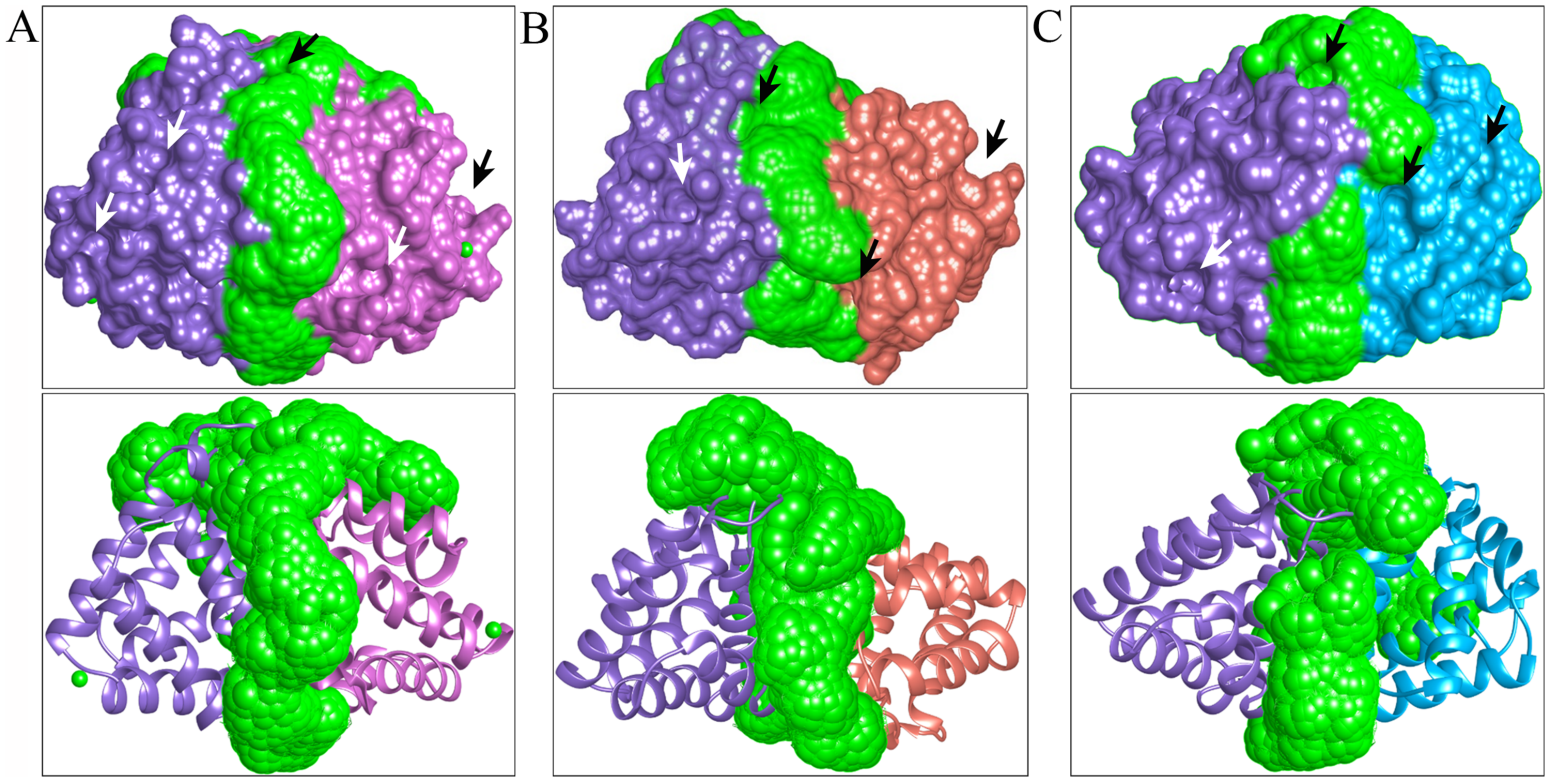
Representation of the surface binding site cleft. (A) The surface and molecular bubble mesh view of the binding site cleft is shown for subunits A (purple) and B (pink) of Fel d 1. (B) The surface and molecular bubble mesh view of the binding site cleft is shown for subunits A of Fel d 1 (purple) and AB dimer (red). (C) The surface and molecular bubble mesh view of binding site cleft were showed for subunit A of Fel d 1 (purple) and AG dimer (light blue). The surface cleft located between two proteins is shown in green in all figures. The arrows represent the surface patches of proteins near the dimer interface.

In Fel d 1, the ligand-binding sites were predicted as two major binding pockets in Fel d 1, and the binding site residues were compare with ABP dimers. The analyses revealed that ABP dimer-binding site residues, such as Cys3, Val10, Phe13, Leu14, Tyr21, Val34, Asn37, Ala38, Ile64, Phe80, Ile115, and Met134, were present in subunits A and B of Fel d 1. Several alternate binding sites were noted between the Fel d 1 and ABP dimers (S3 Table). A similar surface binding site cleft was predicted between the Fel d 1 and ABP dimers, and the central binding cleft is presented as a bubble mesh view with an indication of surface patches (Fig 7A–7C).

### Fel d 1 and ABP similarity evidenced by molecular docking

**Fig 8:**
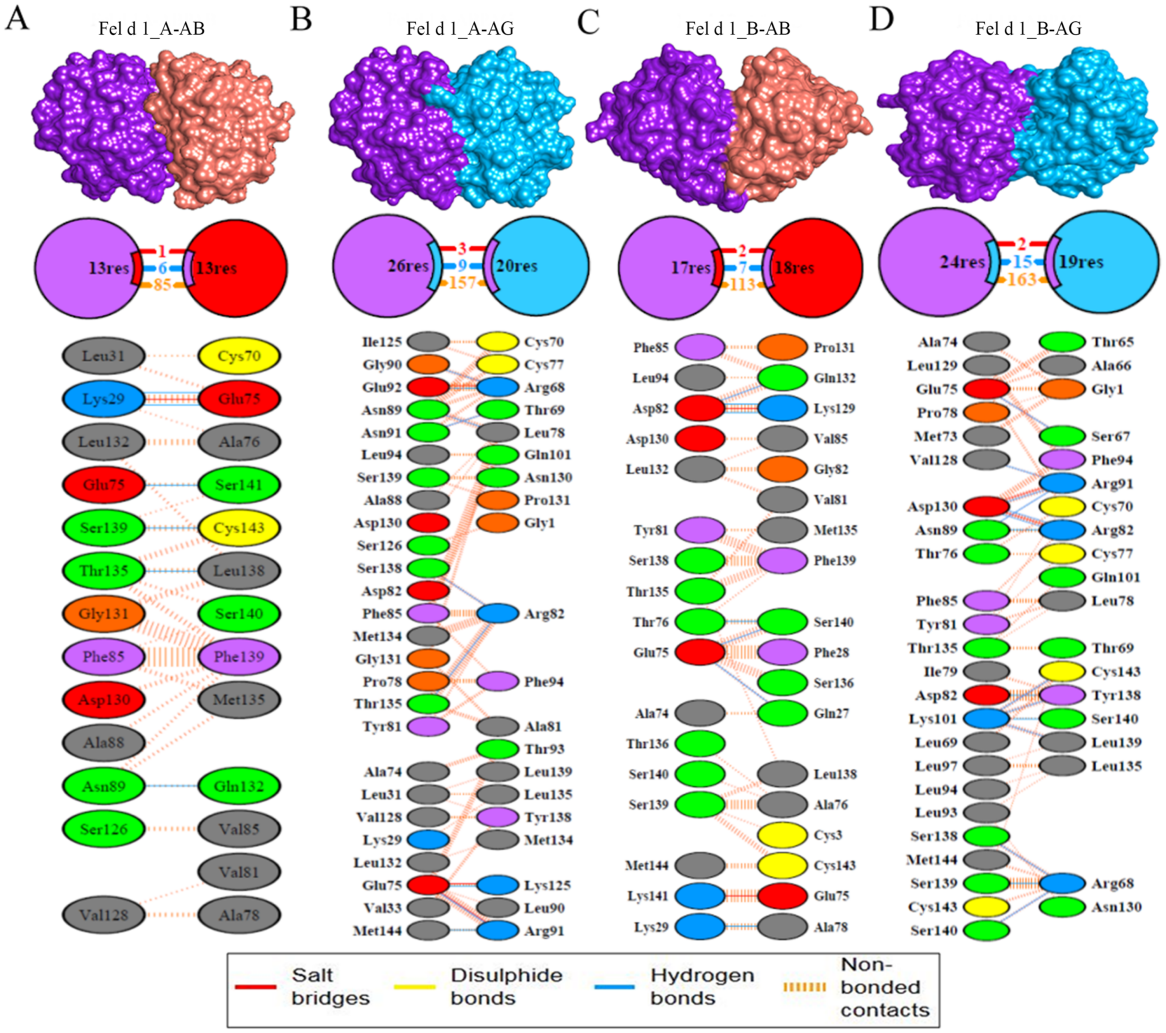
Fel d 1-ABP dimers similarity. (A). Molecular surface view and residue contacts between subunit A of Fel d 1 with the AB dimer. (B). Subunit A of Fel d 1 with the AG dimer. (C). Subunit B of Fel d 1 with the AB dimer. (D). Subunit B of Fel d 1 with AG. All the structural contacts exhibit three different contacts (salt bridges, H-bonds and nonbonded contacts), which are represented with different colors, as indicated in the legend.

The residue surface contacts of ABP dimers were confirmed by comparing the heterodimeric subunits of the Fel d 1 binding sites. The results showed that the binding modes of ABP dimers formed surface contacts with the binding sites of heterodimeric Fel d 1 subunits with the highest rotation and weighted scores (Table 3). Specifically, AG exhibited a greater number of H-bonds with subunit A (9 H-bonds) and B (15 H-bonds) of heterodimeric Fel d 1 than AB. The H-bond residues of AG dimers showed the most prominent position of surface contacts. The numerous hydrophobic and nonbonded contacts were observed between Fel d 1 and AG dimers. Several residue surface contact of ABP dimers were observed at the binding sites of Fel d 1 dimers (Fig 8A–8D). The 5 other modeled ABPA subunits were further used to study Fel d 1 surface structural contacts of Fel d 1. Specifically, AAM08258.1 and AGJ84407.1 showed a greater number of H-bond contacts with subunits A and B of Fel d 1 dimers than the 3 other models. Overall, the binding site residues of heterodimeric Fel d 1 included residues such as Lys29, Glu75, Asn89, and Ser138 in subunits A and Glu75, Asp82, Asn89, Lys101, and Asp130 in subunits B. Therefore, the results suggest that subunit A of Fel d 1 dimers shares its surface binding patches with maximum H-bonds and hydrophobic residues with ABPA (S4 Table).

**Table 3:** The subunits A and B of Fel d 1 and ABP dimer residue contacts were predicted using molecular docking. The residual contacts of AB and AG dimers were denoted. The suitable rotation of binding indicated as rotation of members and weighted score.

Protein-protein	Rotation of members	Weighted score	ClusPro cluster number	Fel d 1-ABP	H-bonds	Hydrophobic and Non-bonded contacts
Fel d 1_A- AB	77	-756.8	3	Lys29-Glu75 Lys29-Glu75 Glu75-Ser141 Asn89-Gln132 Thr135-Leu138 Ser139-Cys143	6	85
Fel d 1_A- AG	156	-890.3	0	Glu75-Lys125 Glu75-Arg91 Asn89-Leu78 Gly90-Arg68 Asn91-Thr69 Glu92-Arg68 Thr135-Arg82 Ser138-Arg82 Met144-Arg91	9	157
Fel d 1_B- AB	173	-755.3	0	Lys29-Ala78 Glu75-Gln27 Glu75-Ser140 Thr76-Ser140 Asp82-Lys129 Asp82-Lys129 Asp82-Gln132	7	113
Fel d 1_B- AG	107	-858.1	1	Glu75-Ser67 Asp82-Tyr138 Asn89-Arg82 Asn89-Arg91 Lys101-Tyr138 Lys101-Leu139 Lys101-Ser140 Lys101-Cys143 Val128-Arg91 Asp130-Arg82 Asp130-Arg82 Asp130-Arg91 Ser138-Arg68 Ser139-Arg68 Ser140-Arg68	15	163

### ABP dimers and steroid interactions

**Fig 9:**
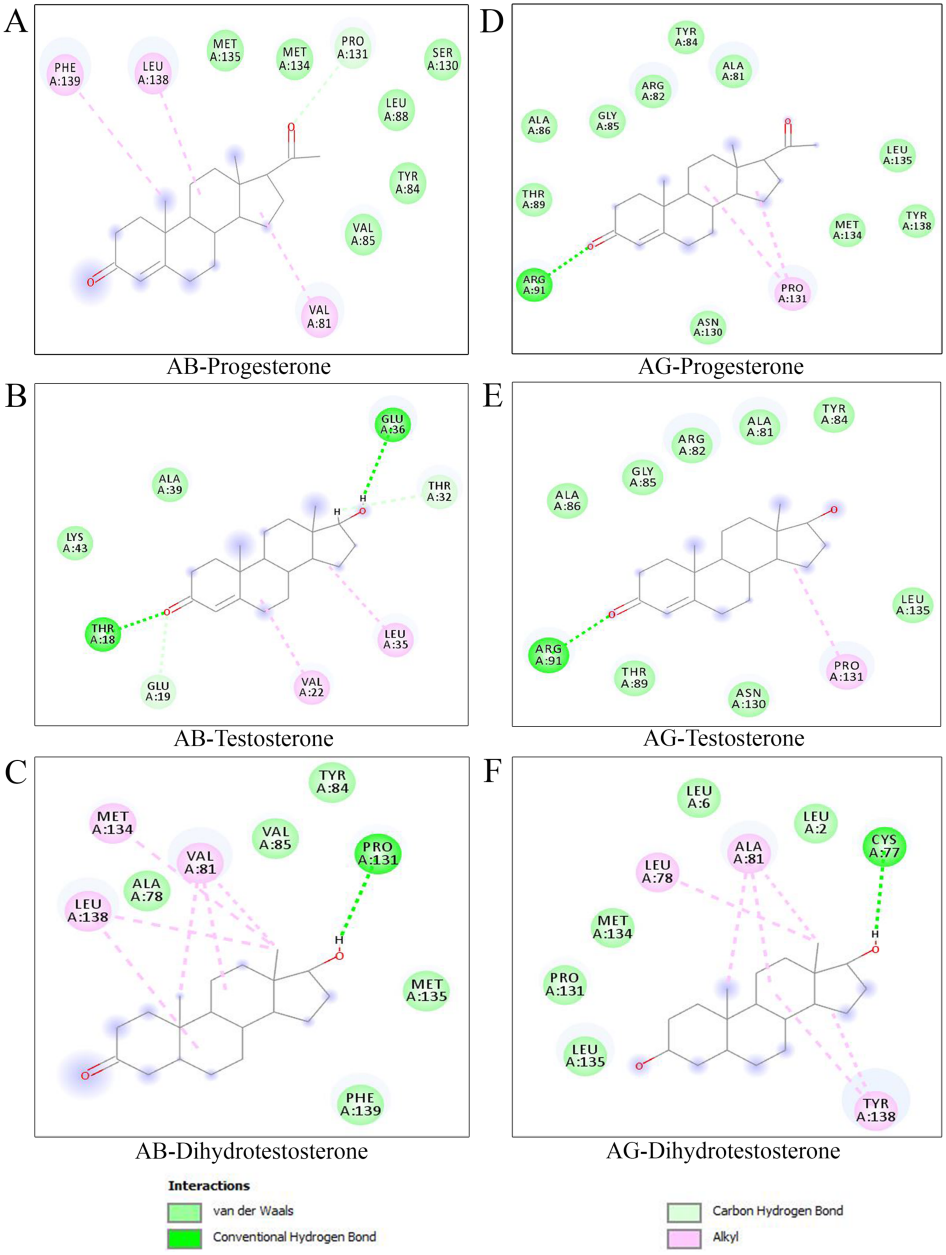
Molecular docking of ABP dimers with steroids. The 2D-residue map of steroid interaction with ABP dimers; the residues within 4.0 Å of the steroids are shown. (A-C). Residue interactions of progesterone (P), testosterone (T), and dihydrotestosterone (DHT) with the AB dimer. (D-F). Residue interactions of progesterone, testosterone, and dihydrotestosterone with the AG dimer. The protein-ligand interactions are depicted as Van der Waals, conventional hydrogen bonds, and alkyl bonds.

A computational evaluation of ABP dimer-steroid interactions was performed using three significant parameters: total energy (ΔG), FullFitness, and H-bond residues. The AB and AG dimers were docked to steroids such as progesterone (P), testosterone (T), and dihydrotestosterone (DHT). The molecular interactions suggest that the AB dimer has excellent binding affinity for ligand T, with a ΔG of -6.8 kcal/mol. Accordingly, AG has a higher binding affinity for ligand DHT than AB, with a ΔG of -6.92 kcal/mol. The AB and AG dimers showed an equivalent ΔG of -6.7 kcal/mol with ligand P (Table 4). Several H-bonds and steric interaction residues were present in the binding site of Fel d 1, and the 2D map of interactions is shown in Fig 9A–9E. Furthermore, the AB dimer preferentially binds to T, and the AG dimer preferentially binds to DHT. Our computational analyses support earlier experimental reports [36] and expand our understanding of these molecular interactions.

**Table 4:** The ABP dimers-steroids residue interactions were predicted using molecular docking.

S. No	Protein Name	Compounds	PubChem ID	Total energy (ΔG) (kcal/mol)	Fullfitness (kcal/mol)	Number of H-bonds	H-bond residues	Van der Waals and alkyl interactions
1	AB	Progesterone	5994	-6.72	-960.27	1	Pro131	Val81, Tyr84, Val85, Leu88, Ser130, Met134, Met135, Leu138, Phe139
2	AG	Progesterone	5994	-6.7	-885.8	2	Arg91	Ala81, Arg82, Tyr84, Gly85, Ala86, Thr89, Asn130, Pro131, Met134, Leu135, Tyr138
3	AB	Testosterone	6013	-6.8	-959.12	2	Thr18, Glu36	Glu19, Val22, Thr32, Leu35, Ala39, Lys43
4	AG	Testosterone	6013	-6.4	-882.9	2	Arg91	Ala81, Arg82, Tyr84, Gly85, Ala86, Thr89, Asn130, Pro131, Leu135
5	AB	Dihydrotestosterone	10635	-6.61	-963.42	1	Pro131	Ala78, Val81, Tyr84, Val85, Met134, Met135, Leu138, Phe139
6	AG	Dihydrotestosterone	10635	-6.92	-901.1	1	Cys77	Leu2, Lue6, Leu78, Ala81, Pro131, Met134, Leu135, Tyr138

ΔG, highest total energy. The residual level of interaction of AB and AG dimers with three steroid compounds. The ΔG of steroid interactions are bold type.

## Discussion

SCGB superfamily members are secreted proteins that are exclusively present in the mammalian lineage [66]. In this superfamily, ABP is considered a proteinaceous pheromone, and its steroid binding ability might play a role in mouse chemical communication [67], [68]. In this study, we performed computational analyses to evaluate the hypothesis that Fel d 1 may exhibit an analogous function to ABP in chemical communication. The clustering of SCGB superfamily proteins supported previously-proposed evolutionary relationships between Fel d 1 and ABP [40]. The two evolutionary trees produced in this work showed that (i) Fel d 1/Mall co-clusters with ABP in various organisms, and (ii) Fel d 1 is a neighbor member to mouse ABP paralogs with the most recent divergence time clades. The results were further explored to reveal that the residue conservation, identical motifs, and binding sites of Fel d 1 were equivalent to those of ABP subunits, thus confirming similar functional features. Hence, ABP subunits were also modeled, and we observed their structural similarity to Fel d 1 based on different topological positions. Moreover, the results showed that Fel d 1 shares many H-bonds with ABP dimers and that the binding sites of AB and AG could be involved in steroid binding, similar to the binding sites of Fel d 1. We also confirmed preferential binding with different affinities for T and DHT of AB and AG, respectively, as has been demonstrated experimentally [36] and corroborated by data from our computational approach.

### Phylogenetic clustering patterns

We observed significant sequence identity of Fel d 1 with ABP and rabbit uteroglobin [33], [35]. The phylogenetic tree indicated that chains 1 and 2 of Fel d 1 are members of the same clusters as UG and MG, respectively, and that the SCGB evolutionary pattern corresponded to the phylogenetic tree of the UG and SCGB family [40], [69], [70]. Determination of the accuracy of identity can enable the discrimination of significant relationships between proteins [71]. We have provided additional gene information for the unrooted phylogenetic tree of the UG family [69] (S4 File). We predicted the evolutionary relationships between Fel d 1/Mall and the ABP subunits using data from 23 mammals. Fel d 1 is essentially the cat version of ABP, where chain 1 of Fel d 1 is the ABPA subunit (cluster 2), and chain 2 of Fel d 1 is the ABPBG (cluster 6) [40]. Thus, the ABP subunits and chains of Fel d 1 are located in clusters 2 and 6, respectively. Additionally, the novel outcomes obtained regarding the molecular similarity of Fel d 1 compared with mouse ABP paralog subunits are noteworthy. The divergence pattern revealed that Fel d 1 is apparently quite closely evolutionarily related to mouse salivary ABP and that these proteins diverged as cocluster and neighbor members. Thus, the preliminary inference is that Fel d 1 diverged with similar divergence time constraints to mouse salivary ABP. This finding justified further analysis of Fel d 1 as a probable steroid-binding protein involved in chemical communication.

### Exploring functional and residue features between the Fel d 1-ABP subunits

Indeed, the hydrophobic sites expressed in Fel d 1 resembled those in ABPA27, and the interior portion corresponds to a hydrophobic region in globular proteins [33], [55]. In addition, we predicted that the maximum conserved sites of Fel d 1 are the same as those of the ABP subunits. Interestingly, when the functional motifs of Fel d 1 were compared with a secretoglobin porcine steroid binding protein (hog pheromaxein) [64] and Fel d 4, a lipocalin kairomone [65], to expose equivalent sites, the MSA suggested that the putative residues of Fel d 1 might exhibit similar functions in chemical communication. We assumed that prominent motifs of the ABP subunits would also observed in Fel d 1. Notably, the Tyr21 residue is conserved in the SCGB superfamily sequence (Fel d 1, ABP, and pheromaxein), and its topological position facilitates ligand binding [14], [72]. Thus, MSA analysis demonstrated that important conserved residues are involved in binding [56].

### Structural modeling and overlap

Protein homology modeling is an essential phase in the determination and comparison of structural alignments with folding patterns between proteins. We modeled ABP subunits according to a previous report [73], and the models were validated using the phi and psi values of Ramachandran plot scores. The automatic homology modeling server used an S2A2 substitution matrix for the detection of homologs through sequence alignment [57]. In particular, it was found that Fel d 1 and ABP both exhibit three cysteine residues that are essential for dimer formation and maintain the monomers in an antiparallel configuration through disulfide bridging [10], [40]. According, to this finding, the conservation of primary and quaternary structure suggest that the genome of the eutherian common ancestor of cats, rodents, and primates possessed a related gene pair [47]. Furthermore, when the AB and AG dimers were superimposed appropriately with Fel d 1, the modeled ABP structure exhibited a similar Cα atom local backbone conformation and shared structural folding with Fel d 1. The structural equivalence alignment and good-quality geometry of the model helped to determine amino acid interactions in the superimposition [59]. The lowest RMSD and maximum GDT_TS are suggestive of similarity to the crystal structure of Fel d 1. The present results confirmed that structural relationships and functional similarities in the SCGB family might be associated with the binding of small hydrophobic ligands.

### Simulation and binding site analysis

Subsequently, the dynamic simulation of ABP subunits showed maximum flexibility in the central binding region and the entrance of binding pocket residues. The RMSD and RMSF plot depicted similarities in folding dynamics. The results reinforced the notion that biological functions of proteins can be predicted based on protein structural conformations and flexibility [60]. The surface patches were predicted based on Cα atom coordinates, and the circular patches were observed to be contiguous on the protein surface [74]. Another important finding regarding Fel d 1 chains and ABP subunits and dimers is that when they were used to predict major binding sites, the putative cavities of Fel d 1 exhibited a similar residue pattern to those of ABP subunits, which are highlighted in the binding site table (S3 Table).

### Molecular docking analysis

In general, the AB dimers are found in mouse saliva at nearly equal-molar levels to AG dimers [36]. Prediction of the binding interaction based on sites of positive selection sites (ω^+^) and mapped to Fel d 1 which showed a majority of ω^+^ sites on the surface and an absence in the internal cavity of the dimers [48]. In support of this previous report, the AG dimers showed a greater number of H-bonds and hydrophobic interactions with Fel d 1 than the AB dimers in the present study. Similar structural contacts were found at the surface binding sites of Fel d 1 with the AB and AG dimers, with different docking positions. Likewise, the AB and AG dimers showed a greater number of molecular interactions with T and DHT, respectively, with different affinities. Additionally, the AB and AG dimers exhibited equivalent interactions with P. The differential affinities of the ABP dimers suggest that stimulus preferences are involved in adaptive evolution and influence mate choice [73]. Recently, Chung et al. (2017) demonstrated in a knockout study that mice are capable of detecting the presence/absence of ABP in saliva from the opposite sex, supporting a role of ABP in intraspecific chemical communication [44]. In addition, previous studies demonstrated a close association of Fel d 1 production areas and pheromone releasing areas in cats and showed that cat sex and behavior affect the immunological properties of Fel d 1, which suggested that Fel d 1 could be involved in feline chemical communication [18], [34]. Therefore, the computational outcome was corroborated by earlier experimental findings [36]. Indeed, we have highlighted additional computational evidence to support the hypothesis of a functional role of Fel d 1 in chemical communication.

### Structural and functional similarities between Fel d 1 and ABP

Based on our computational analysis, we identified similar motifs and conserved residues of Fel d 1 and ABP. The conserved residues were classified as functionally exposed and structurally buried residues in both proteins. We observed many identical binding site residues in Fel d 1 and ABP, which led us to the identification of structural superimposition and surface binding site cleft prediction in their structural models. In addition, we predicted H-bonds and hydrophobic contacts between Fel d 1 and ABP dimers. The described protein-protein analysis actively revealed several significant bonds. Furthermore, we performed ABP dimer-pheromone docking analysis. The AB and AG dimers showed different binding affinities and residue interactions with steroids. The results confirmed the earlier experimental hypothesis that ABP dimers exhibit different affinities with steroids [73]. However, the structural similarities of surface patches implied that they might interact with similar semiochemical cues with similar hydrophobic binding pockets. Overall, these structural similarities could lead to the elucidation of functional significance in both proteins.

In conclusion, this work is the first report of a computational analysis revealing the structural resemblance, molecular protein-protein docking, and preferential binding of steroids between Fel d 1 and ABP dimers. While this bioinformatic approaches is not fully demonstrative and must be confirmed via other analysis (such as behavioral studies or wet-lab approaches such as *in vitro* ligand-binding assays), it provides insightful information that could support the elucidation of the biological role of Fel d 1 and help to design appropriate wet-lab approaches. Indeed, the study corroborates that Fel d 1 and ABP exhibit similar structural and functional characteristics, and the both could be involved in semiochemical transport/processing in intraspecies communications. Therefore, it will be interesting to elucidate the pattern of the stability of ABP dimers interacting with Ca^2+^ metal ions, as reported for Fel d 1 [23], [75]. Additionally, it is important to examine the structural conformations and folding patterns of Fel d 1 and ABP with pheromones using molecular dynamic simulations. Ultimately, chemical identification of Fel d 1 endogenous ligands of Fel d 1 would provide additional strong evidence of the role of Fel d 1 in chemical communication in cats. Therefore, our ongoing studies are aimed at analyzing and confirming the biological function of the steroid and pheromone binding of Fel d 1, using wet-lab methods.

## Supporting information

S1 Fig Curved and circular view of selected members of the SCGB superfamily phylogeny. (A) The curve view phylogeny was constructed using selective members from SCGB superfamily. (B) The Circular evolutionary tree is projecting deviation between SCGB. The tree was estimated using a scale bar of 0.2 amino acid substitution matrices per site.
(PDF)

S2 Fig Evolutionary tree of Fel d 1/Mall chains and ABP subunits. (A) Phylogenetic tree of Fel d 1-ABP was constructed using branch length. (B) Radial shape tree projecting deviation between Fel d 1/Mall and ABP. (C) Circular representation of unrooted evolutionary tree of Fel d 1/Mall (Red) and ABP (Brown). The Fel d 1/Mall-ABP was estimated using a scale bar of 0.5 amino acid substitution matrices per site.
(PDF)

S3 Fig Percentage sequence identity matrix representing the average identity between clusters 2 and 6 with a color scale of percent identities. The PIM values were shown as 0–100% identity of sequences alignment with five different colors on a scale from 0–10 (light blue), 10.1–30 (dark blue), 30.1–50 (light yellow), 50.1–70 (dark yellow), 70.1–100 (red).
(PDF)

S4 Fig Divergence time tree of Fel d 1 chains with mouse ABP paralogs. The molecular divergence time tree was estimated for Fel d 1 chains with 28 ABP paralog dataset. The divergence of both proteins was showed most common divergence lineage. The Fel d 1 sequence are similarly coclustered with ABP subunits. The scale bar in million years ago (MYA).
(PDF)

S5 Fig Hydrophobic site prediction between chains of Fel d 1-ABP subunits without signal peptides and Fel d 1 dimer. The similar hydrophobic plot was predicted based on alignment of ABP subunits towards chains of Fel d 1. The low score of hydrophobicity was marked in Fel d 1 dimer interface.
(PDF)

S6 Fig Sequence alignment of chains of Fel d 1 with ABP subunits and Fel d 1-ABP dimers. Sequence similarity of chains of Fel d 1 with (A) ABPA27, (B) ABPBG27, (C) ABPBG26, and (D)Fel d 1 dimer with AB-AG dimers. The sequence alignment of Fel d 1-ABP subunits pertained highest number of identical and semi identical residues are indicated as (_*_) and (:) respectively.
(PDF)

S7 Fig Sequence alignment of chains of Fel d 1 with pheromaxein subunits and Fel d 1 dimer with Fel d 4. Sequence similarity of chains of Fel d 1 with (A) PheromaxeinC (B) PheromaxeinA and (C) Fel d 1 dimer with Fel d 4. The results showed highest number of identical and semi identical residues are indicated as (_*_) and (:) respectively.
(PDF)

S8 Fig Homology modeling. (A) The selected subunits of ABPA27 (pink) and ABPBG27 (red) were modeled and made as a AB dimer (surface view). (B) The selected subunits of ABPA27 (pink) and ABPBG26 (light blue) were modeled and made as a AG dimer (surface view). The conserved residues were denoted as blue molecular sticks in all ABP subunits. (C) Structural validation of ABPA27, (D) ABPBG27 and E) ABPBG26 by Ramachandran Plot.
(PDF)

S9 Fig Structural deviation and fluctuation of ABP subunit protein residues in a dynamic simulation. (A) The plot showed the RMSD difference between ABP subunits. (B) The plot shows RMS fluctuation in ABP subunit residues. The results showed ABPA had less fluctuations with a maximum cluster density of 215.2 and an average RMSD cluster of 1.1 Å.
(PDF)

S1 File The SCGB protein sequence dataset. (XLSX)

S2 File List of 64 ABP paralogs with pseudogene indications [ref.^8^]. (XLSX)

S3 File Gene list from 3 UG protein clusters. (XLSX)

S4 File Gene list from the unrooted phylogeny of the UG family [ref. 69]. (XLSX)

S1 Table Geographical origin of species and short terms for zoological names. (DOCX)

S2 Table List of 28 ABP paralogs with proteins IDs. The ABP paralog dataset was curated without pseudogenes. The list of paralogs was collected from earlier report [8] and additionally added protein name, protein ID, strain, conserved protein domain ID.
(DOCX)

S3 Table Comparison of binding site profiles between Fel d 1 and ABP dimers. The similar binding site residues were highlighted as bold in Fel d 1 and ABP dimers. Also, the AB and AG dimers have similar alternate residues in the Fel d 1 dimer binding pockets.
(DOCX)

S4 Table Predicted structural contacts between subunits A and B of Fel d 1 with subunits of ABPA. The H-bond with positions are tabulated based on members.
(DOCX)
