# Supplementary material for: Another cat and mouse game: Deciphering the evolution of the SCGB superfamily and exploring the molecular similarity of major cat allergen Fel d 1 and mouse ABP using computational approaches
Source: PLoS One. 2018 May 17;13(5):e0197618. doi: 10.1371/journal.pone.0197618 (PMC5957422; doi:10.1371/journal.pone.0197618)
Supplement: S1 Fig — (A) The curve view phylogeny was constructed using selective members from SCGB superfamily. (B) The Circular evolutionary tree is projecting deviation between SCGB. The tree was estimated using a scale bar of 0.2 amino acid substitution matrices per site. (PDF) [file pone.0197618.s001.pdf]

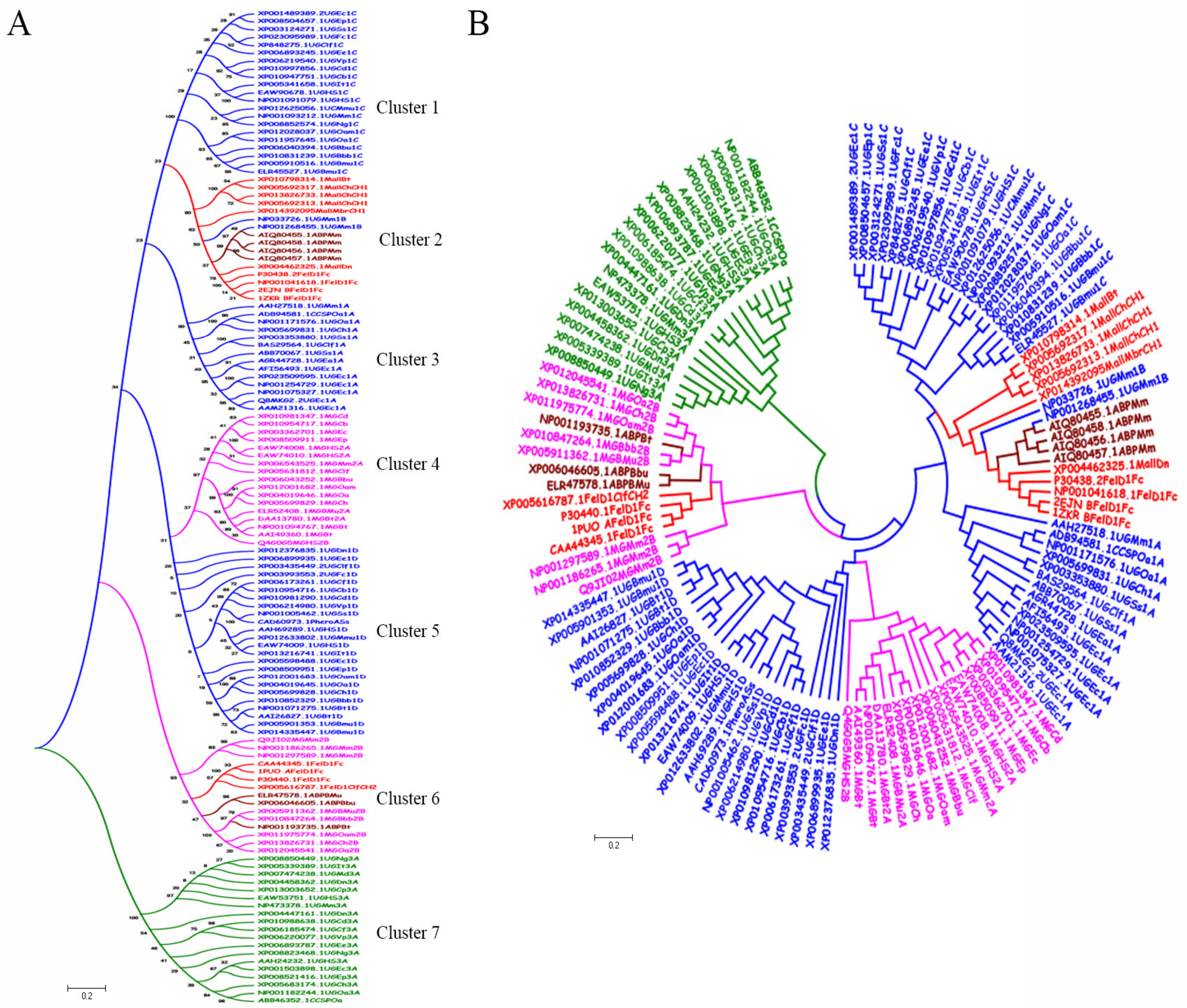
A
B
Cluster 1
Cluster 2
Cluster 3
Cluster 4
Cluster 5
Cluster 6
Cluster 7
0.2
0.2

.
