# Supplementary figures and images for: Another cat and mouse game: Deciphering the evolution of the SCGB superfamily and exploring the molecular similarity of major cat allergen Fel d 1 and mouse ABP using computational approaches
Source: PLoS One. 2018 May 17;13(5):e0197618. doi: 10.1371/journal.pone.0197618 (PMC5957422; doi:10.1371/journal.pone.0197618)

A

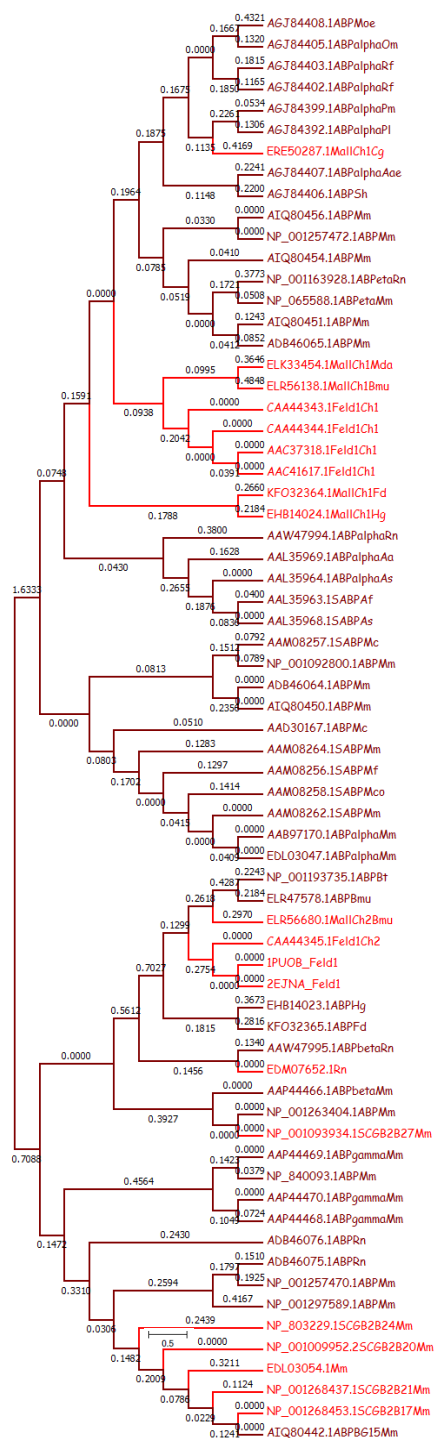

Cluster 2

Cluster 6

B

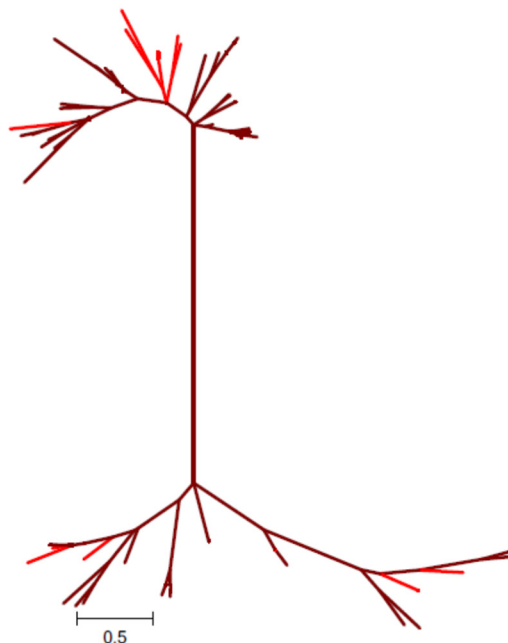

C

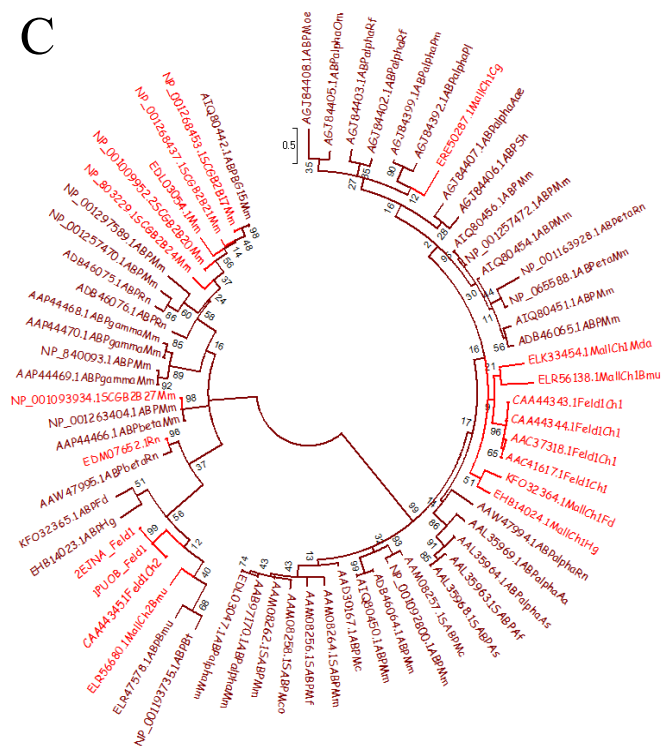

Supplement: S2 Fig — (A) Phylogenetic tree of Fel d 1-ABP was constructed using branch length. (B) Radial shape tree projecting deviation between Fel d 1/Mall and ABP. (C) Circular representation of unrooted evolutionary tree of Fel d 1/Mall (Red) and ABP (Brown). The Fel d 1/Mall-ABP was estimated using a scale bar of 0.5 amino acid substitution matrices per site. (PDF) [file pone.0197618.s002.pdf]

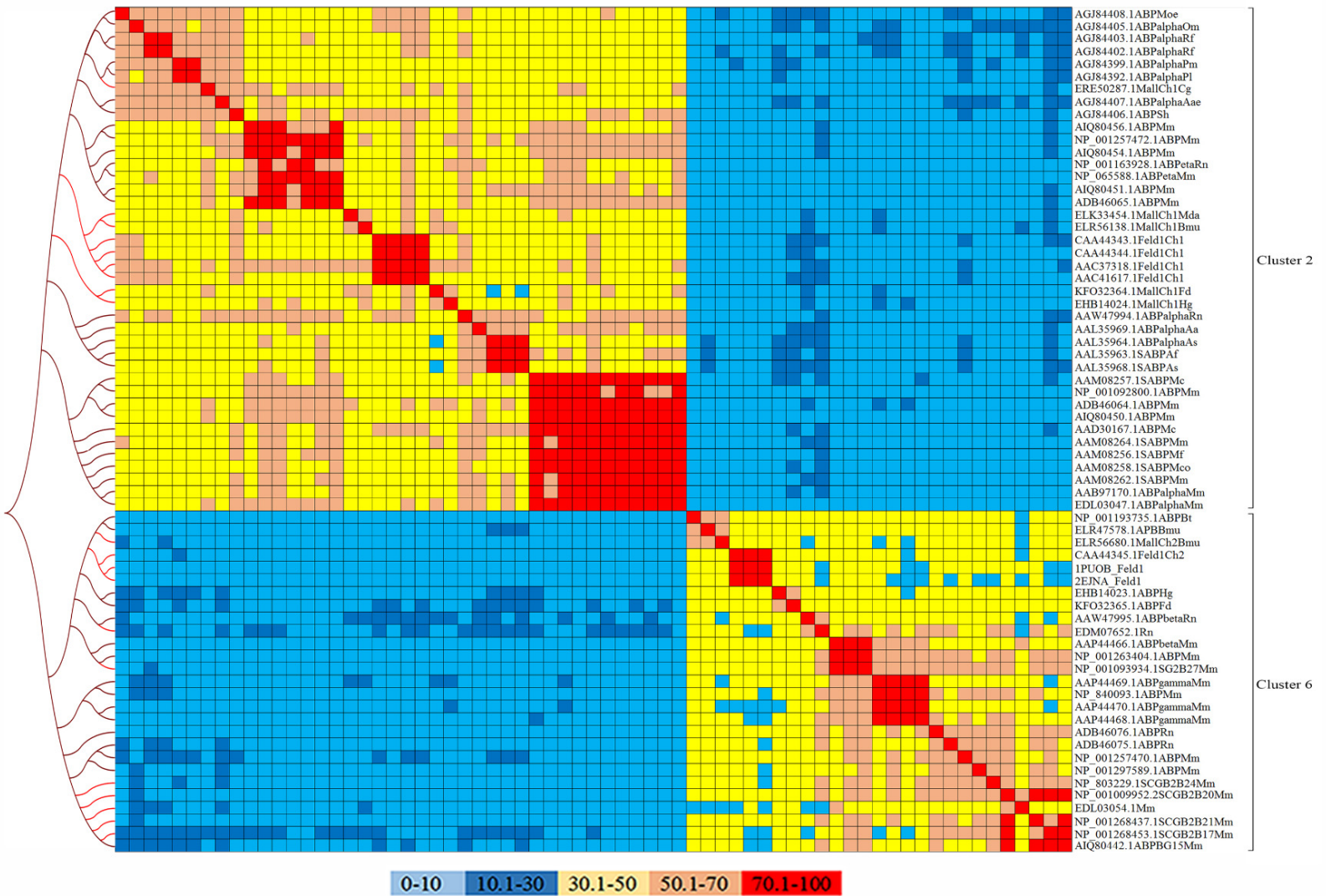

Supplement: S3 Fig — The PIM values were shown as 0–100% identity of sequences alignment with five different colors on a scale from 0–10 (light blue), 10.1–30 (dark blue), 30.1–50 (light yellow), 50.1–70 (dark yellow), 70.1–100 (red). (PDF) [file pone.0197618.s003.pdf]

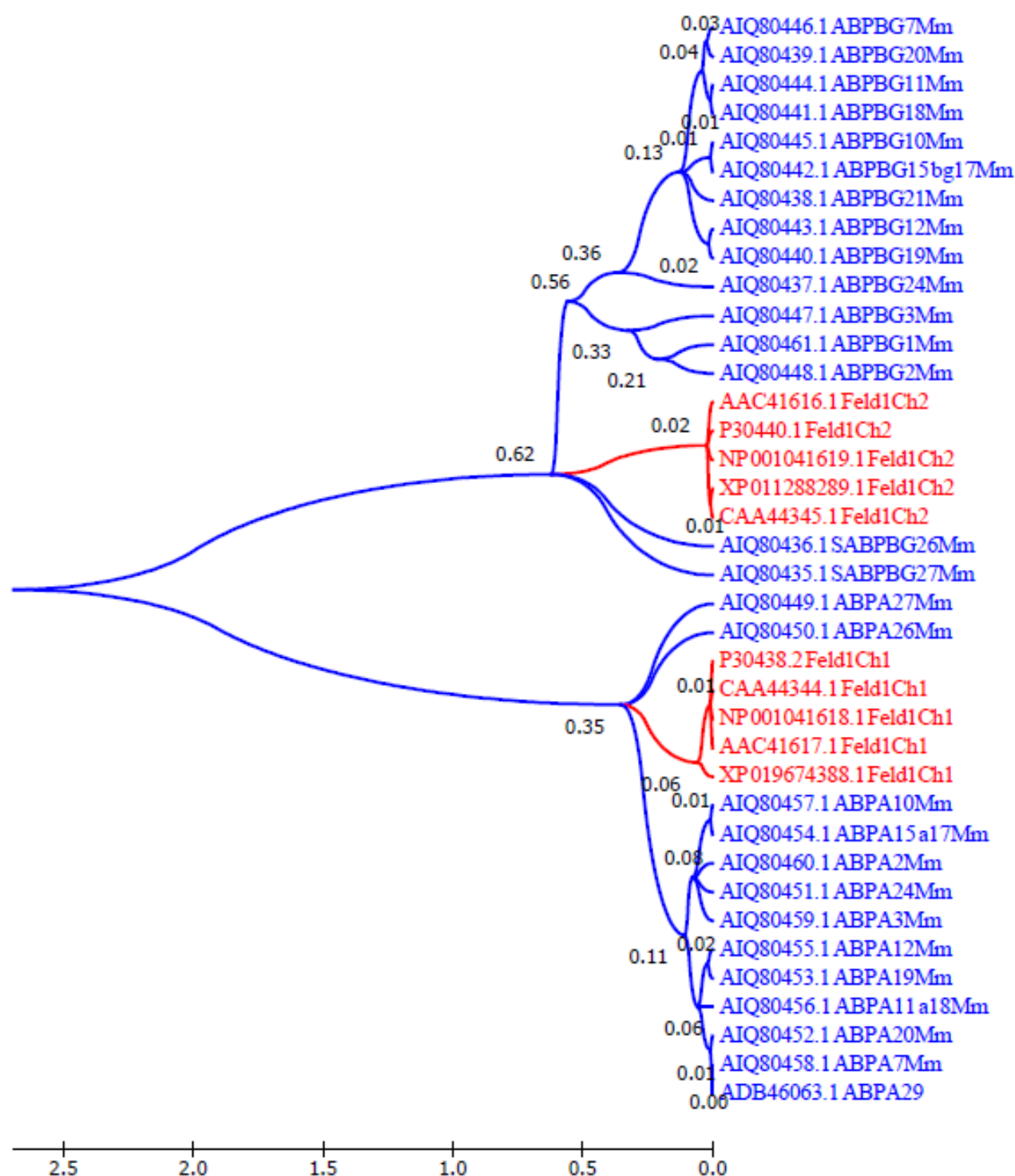

Supplement: S4 Fig — The molecular divergence time tree was estimated for Fel d 1 chains with 28 ABP paralog dataset. The divergence of both proteins was showed most common divergence lineage. The Fel d 1 sequence are similarly coclustered with ABP subunits. The scale bar in million years ago (MYA). (PDF) [file pone.0197618.s004.pdf]

A

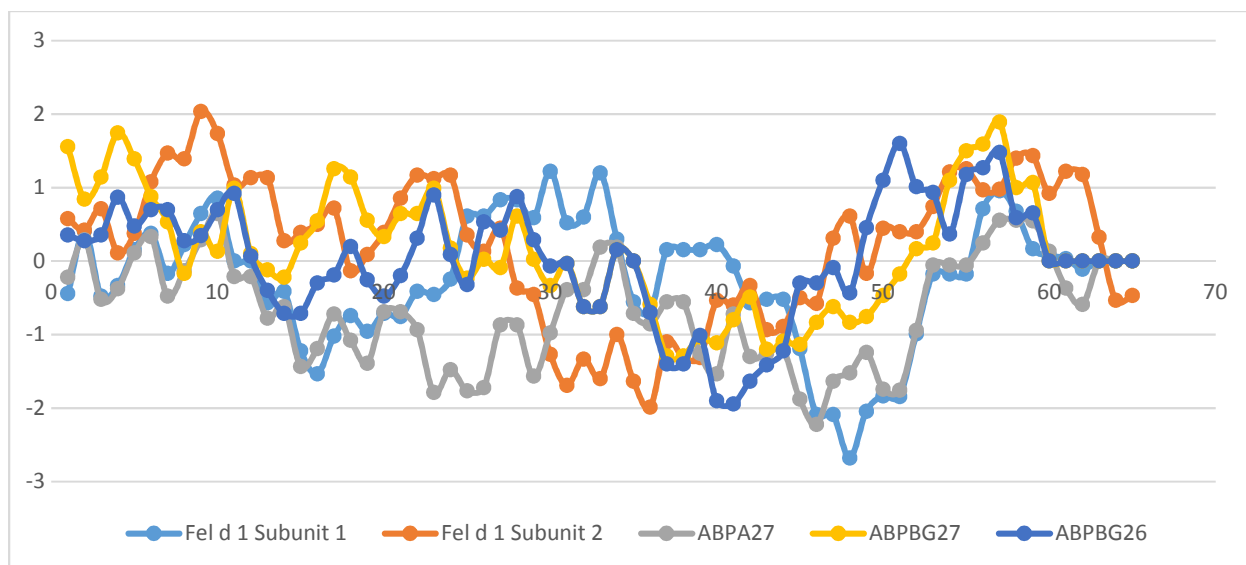

B

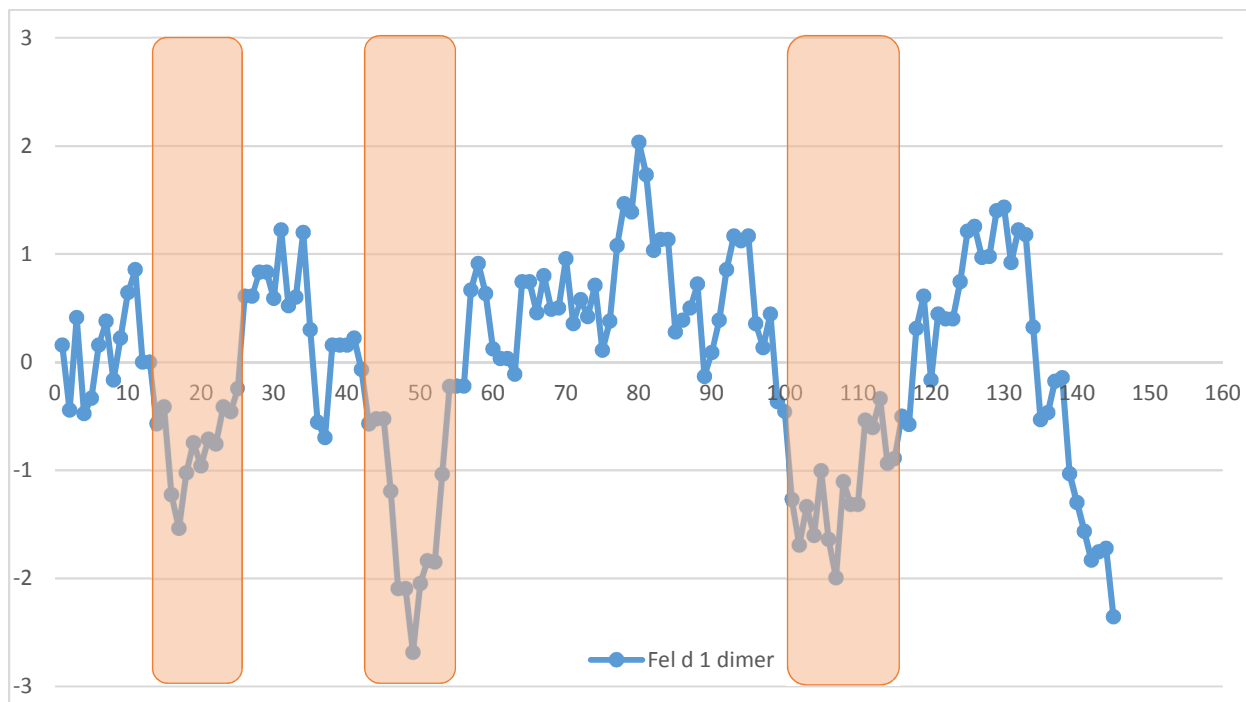

Supplement: S5 Fig — The similar hydrophobic plot was predicted based on alignment of ABP subunits towards chains of Fel d 1. The low score of hydrophobicity was marked in Fel d 1 dimer interface. (PDF) [file pone.0197618.s005.pdf]

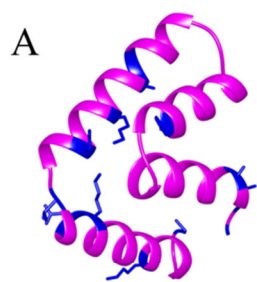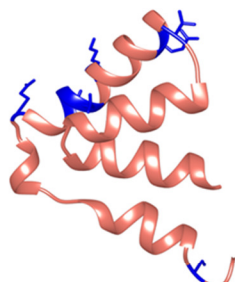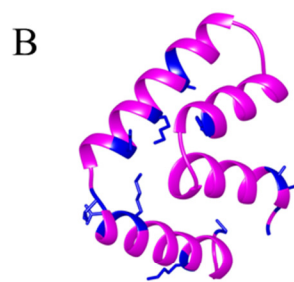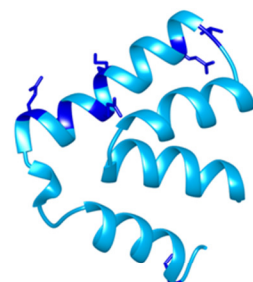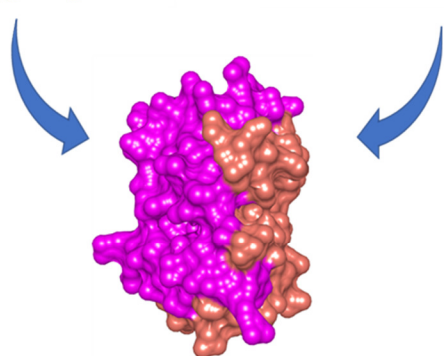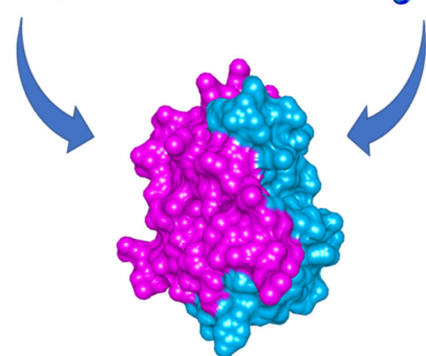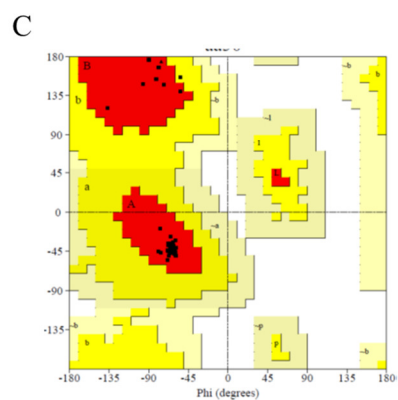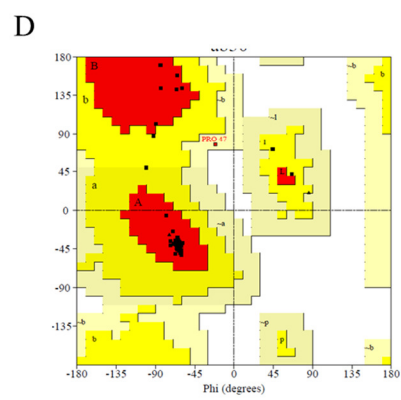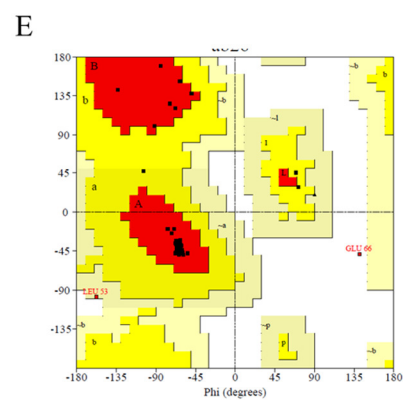

Supplement: S8 Fig — (A) The selected subunits of ABPA27 (pink) and ABPBG27 (red) were modeled and made as a AB dimer (surface view). (B) The selected subunits of ABPA27 (pink) and ABPBG26 (light blue) were modeled and made as a AG dimer (surface view). The conserved residues were denoted as blue molecular sticks in all ABP subunits. (C) Structural validation of ABPA27, (D) ABPBG27 and E) ABPBG26 by Ramachandran Plot. (PDF) [file pone.0197618.s008.pdf]

A

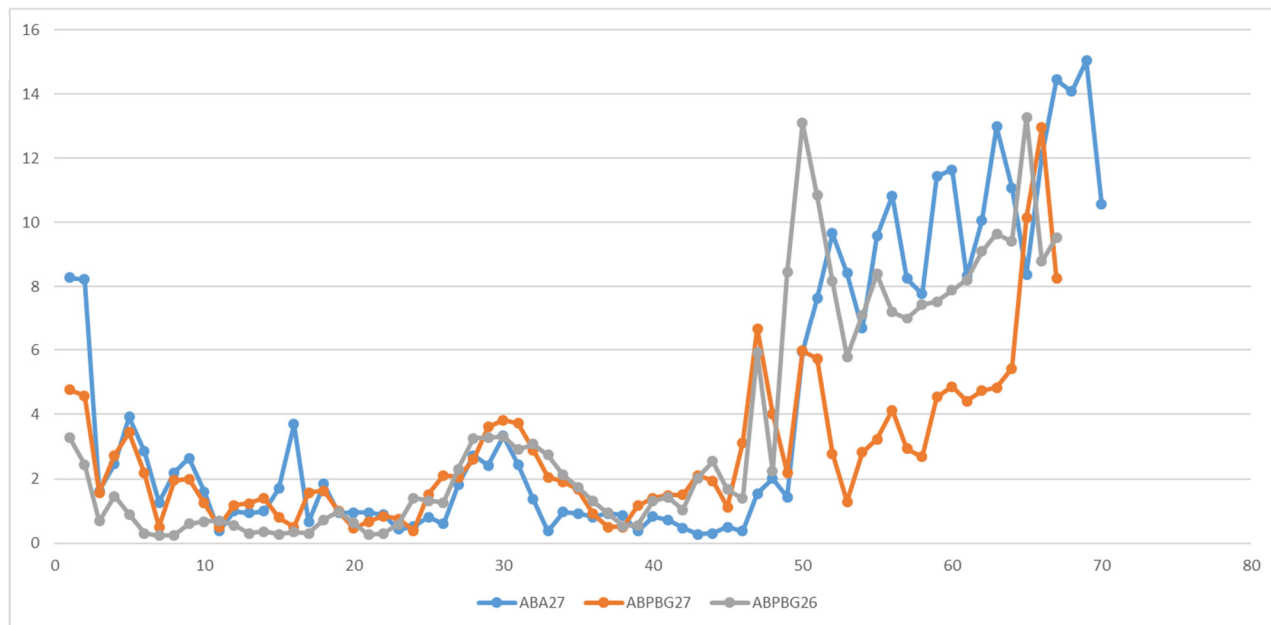

B

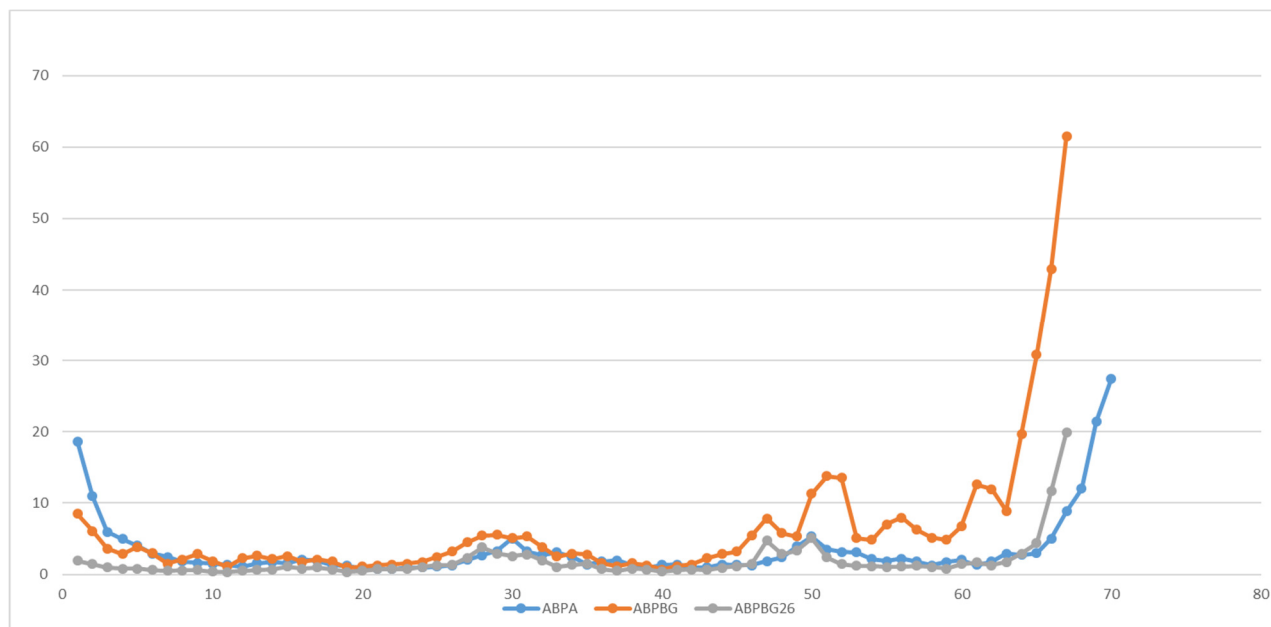

Supplement: S9 Fig — (A) The plot showed the RMSD difference between ABP subunits. (B) The plot shows RMS fluctuation in ABP subunit residues. The results showed ABPA had less fluctuations with a maximum cluster density of 215.2 and an average RMSD cluster of 1.1 Å. (PDF) [file pone.0197618.s009.pdf]
