# Supplementary material for: Another cat and mouse game: Deciphering the evolution of the SCGB superfamily and exploring the molecular similarity of major cat allergen Fel d 1 and mouse ABP using computational approaches
Source: PLoS One. 2018 May 17;13(5):e0197618. doi: 10.1371/journal.pone.0197618 (PMC5957422; doi:10.1371/journal.pone.0197618)
Supplement: S6 Fig — Sequence similarity of chains of Fel d 1 with (A) ABPA27, (B) ABPBG27, (C) ABPBG26, and (D)Fel d 1 dimer with AB-AG dimers. The sequence alignment of Fel d 1-ABP subunits pertained highest number of identical and semi identical residues are indicated as (*) and (:) respectively. (PDF) [file pone.0197618.s006.pdf]

A

```
Feld1_Ch1       EICPAVKRDVDLFLTGTPDEYVEQVAQYKALPVVLENARILKNCVDAKMTEEDKENALSV
ABPA27          GLCPALQRKVDLFLNGTTEEYVEYLKQFNENTKVLENAANIKKCSDRTLTEEDKAQATSL
                 :***::*.*****.** :**** : *::    *****  :*:* * .:***** :* *:

Feld1_Ch1       LDKIYTSPLC
ABPA27          INKITASRTC
                ::** :*  *
```

B

```
Feld1_Ch2       VKMAETCPIFYDVFFAVANGNELLLDLSLTKVNATEPERTAMKKIQDCYVENGLISRVLD
ABPBG27         ------CAPFVGAYVKILGGNRLALNAYLSMFQATAAERVAFEKIQDCFNEEPLTTKLKS
                      *  * ..:. : .**.* *:  *: .:**  **.*::*****: *: * ::: .

Feld1_Ch2       GLVMTTISSSKDC
ABPBG27         PQIMMSILFSSEC
                  :* :*  *.:*
```

C

```
Feld1_Ch2       VKMAETCPIFYDVFFAVANGNELLLDLSLTKVNATEPERTAMKKIQDCYVENGLISRVLD
ABPBG26_Mm      ------CLSFARTYGAILTLRRTFLHGDLSQFYATVAERVAFEKIQDCFREEGQKTIILN
                      *  *  .:  *: . .. :*. .*::. **  **.*::*****: *:*  :  :*:

Feld1_Ch2       GLVMTTISSSKDC
ABPBG26_Mm      PQIMLSLYLSPEC
                  :* ::  * :*
```

D

```
2EJN_A          MEICPAVKRDVDLFLTGTPDEYVEQVAQYKALPVVLENARILKNCVDAKMTEEDKENALS
AGdimer         -GLCPALQRKVDLFLNGTTEEYVEYLKQFNENTKVLENAANIKKCSDRTLTEEDKAQATS
ABdimer         -GLCPALQRKVDLFLNGTTEEYVEYLKQFNENTKVLENAANIKKCSDRTLTEEDKAQATS
                  :***::*.*****.** :**** : *::    *****  :*:* * .:***** :* *

2EJN_A          LLDKIYTSPLCVKMAETCPIFYDVFFAVANGNELLLDLSLTKVNATEPERTAMKKIQDCY
AGdimer         LINKITASRTC------CLSFARTYGAILTLRRTFLHGDLSQFYATVAERVAFEKIQDCF
ABdimer         LINKITASRTC----EACAPFVGAYVKILGGNRLALNAYLSMFQATAAERVAFEKIQDCF
                *::** :*  *      *  *  .:  :    ..  *.  *: .  **  **.*::*****:

2EJN_A          VENGLISRVLDGLVMTTISSSKDCMGEHHHHHH
AGdimer         REEGQKTIILNPQIMLSLYLSPEC---------
ABdimer         NEEPLTTKLKSPQIMMSILFSSEC---------
                 *:   : : .  :* ::  *  :*
```
