# Supplementary material for: Another cat and mouse game: Deciphering the evolution of the SCGB superfamily and exploring the molecular similarity of major cat allergen Fel d 1 and mouse ABP using computational approaches
Source: PLoS One. 2018 May 17;13(5):e0197618. doi: 10.1371/journal.pone.0197618 (PMC5957422; doi:10.1371/journal.pone.0197618)
Supplement: S7 Fig — Sequence similarity of chains of Fel d 1 with (A) PheromaxeinC (B) PheromaxeinA and (C) Fel d 1 dimer with Fel d 4. The results showed highest number of identical and semi identical residues are indicated as (*) and (:) respectively. (PDF) [file pone.0197618.s007.pdf]

A

```
Feld1_Ch1                   MKGACVLVLLWAALLLISGGNCEICPAVKRDVDLFLTGTPDEYVEQVAQYKALPVVLENA
NP_001116633.1_PheroC       MKLAMVLMLVALPVYCSAGSGCSYLERVISDTSD-SSVTTDVYLASLQEYISSDDTTQAI
                            ** * **:*:   :   :*..*.    *  *..   : * * *: .: :* :   . :

Feld1_Ch1                   RILKNCVDAKMTEEDKENALSVLDKIYTSPLC---
NP_001116633.1_PheroC       KELRECFLK-QSEETLENFSVFMQVTYNSKLCAAF
                            : *::*.    :**  **   .::  *.* **
```

B

```
Feld1_Ch2                   MRGALLV----LALLVTQALGVKMAETCPIFYDVFFAVANGNELLLDLSLTKVNATEPER
CAD60973.1_pheroA           MRLSLTVLLVTLALCCYEAHGI----VCRALVKEFSAFLWKPDEIYKPELELFGAPPEAV
                            ** :* *    ***   :* *:    .*  : . * *.    : : . .*  ..*

Feld1_Ch2                   TAMKKIQDCYVENGLISRVLDGLVMTTISSSKDCMGEAVQNTVEDLKLNTLGR---
CAD60973.1_pheroA           DAKMKVKQCANGISFKKKILLTKTLVEI-LVKKCGFEDVKTLFPDISLGLSASVFK
                             *  *:::*    .: .::*   .:. *   *.*  * *:. . *:.*.  .
```

C

```
2EJN_A_FelD1Fc              ----------MEICPAVKR-----DVD----------LFLTGTPDEYVEQVAQYKALPVV
Feld4Fc                     MKLLLLCLGLILVCAHEEENVVRSNIDISKISGEWYSILLASDVKE---KIEENGSMRVF
                                      : :*   :.     ::*          ::*:.  .*   :: :  :: *.

2EJN_A_FelD1Fc              LENARILK--------------NCVDAKMTEE-----------DKENALSLLDKIYTSPL
Feld4Fc                     VEHIKALDNSSLSFVFHTKENGKCTEIFLVADKTKDGVYTVVYDGYNVFSIVETVYDEYI
                            :*: : *.              :*.:  :. :           *  *.:*:::.:* . :

2EJN_A_FelD1Fc              CV---KMAETCPIFYDVFFAVANGNELLLDLSLTKVNATEPERTAMKKIQDCY----VEN
Feld4Fc                     LLHLLNFDKTRPFQ------------------LVEFYAREPDVS--QKLKEKFVKYCQEH
                             :   :: :* *:                   *.:. * **: :  :*::: :     *:

2EJN_A_FelD1Fc              GLISRVLDGLVMTTISSSKDCMGEHHHHH---H
Feld4Fc                     GIVN-ILD---LTEVDRCLQARGSEVAQDSSVE
                            *::. :**   :* :. . :. *..  :.   .
```
