# Supplementary material for: Another cat and mouse game: Deciphering the evolution of the SCGB superfamily and exploring the molecular similarity of major cat allergen Fel d 1 and mouse ABP using computational approaches
Source: PLoS One. 2018 May 17;13(5):e0197618. doi: 10.1371/journal.pone.0197618 (PMC5957422; doi:10.1371/journal.pone.0197618)
Supplement: S1 Table — (DOCX) [file pone.0197618.s014.docx]

| S. No | Zoological name | Short term | Common name | Found |
| --- | --- | --- | --- | --- |
| 1 | *Microtus oeconomus* | Moe | Tundra vole | North America |
| 2 | *Oligoryzomys microtis* | Om | Small-eared pygmy rat | South America; Western Brazil |
| 3 | *Reithrodontomys fulvescens* | Rf | Fulvous harvest mouse | El Salvador, Guatemala, Honduras, Mexico, Nicaragua, And United States |
| 4 | *Peromycus maniculatus* | Pm | Deer mouse | North America |
| 5 | *Peromycus leucopus* | Pl | White-footed mouse | North America From Ontario, Quebec, Labrador |
| 6 | *Cricetulus griseus* | Cg | Chinese hamster | Northern China and Mongolia |
| 7 | *Akodon aerosus* | Aae | Highland grass mouse | Eastern Ecuador Through Peru Into Central Bolivia |
| 8 | *Sigmodon hispidus* | Sh | Hispid cotton rat | South America, Central America, And Southern North America |
| 9 | *Mus musculus* | Mm | House mouse | All Over the World |
| 10 | *Rattus norvegicus* | Rn | Brown rat | Norway Rat |
| 11 | *Myotis davidii* | Mda | Vesper bat | Hebei, Hubei, Inner Mongolia, Shaanxi, Shanxi And Hong Kong |
| 12 | *Bos mutus* | Bmu | Yak | Himalaya Region of Southern Central Asia |
| 13 | *Felis domesticus* | Fel d 1 | House cats | United States |
| 14 | *Fukomys damarensis* | Fd | Damaraland mole-rat | Southern Africa |
| 15 | *Heterocephalus glabber* | Hg | Naked mole-rat | East Africa |
| 16 | *Apodemus agaricus* | Aa | Striped field mouse | Eastern Europe To Eastern Asia |
| 17 | *Apodemus sylvaticus* | As | Wood mouse | From Europe And Northwestern Africa |
| 18 | *Apodemus falvicollis* | Af | Yellow-necked mouse | South China Field Mouse |
| 19 | *Mus cervicol* | Mc | Fawn-colored mouse | Cambodia, India, Possibly Indonesia, Laos, Myanmar, Nepal, Thailand, And Vietnam |
| 20 | *Mus famulus* | Mf | Servant mouse | Only in India |
| 21 | *Mus cookii* | Mco | Cook's mouse | China, India, Laos, Myanmar, Nepal, Thailand, And Vietnam |
| 22 | *Bos taurus* | Bt | Cattle | Europe, North Africa, And Much of Asia |
| 23 | *Microcebus murinus* | Mmu | Gray mouse lemur | Island of Madagascar |
