# Supplementary material for: Another cat and mouse game: Deciphering the evolution of the SCGB superfamily and exploring the molecular similarity of major cat allergen Fel d 1 and mouse ABP using computational approaches
Source: PLoS One. 2018 May 17;13(5):e0197618. doi: 10.1371/journal.pone.0197618 (PMC5957422; doi:10.1371/journal.pone.0197618)
Supplement: S2 Table — The ABP paralog dataset was curated without pseudogenes. The list of paralogs was collected from earlier report [8] and additionally added protein name, protein ID, strain, conserved protein domain ID. (DOCX) [file pone.0197618.s015.docx]

| S. No | Gene Name | Gene Bank Account Number | Chromosome number | Protein Name | Protein ID | Strain | Conserved protein domain ID |
| --- | --- | --- | --- | --- | --- | --- | --- |
| 1 | B6_bg1 | KM014083 | 7 | ABPBG1 | AIQ80461.1 | C57BL/6 | CDD:286352 |
| 2 | B6_a2 | KM014043 | 7 | ABPA2 | AIQ80460 | C57BL/6 | CDD:279445; CDD:238346 |
| 3 | B6_bg2 | KM014055 | 7 | ABPBG2 | AIQ80448.1 | C57BL/6 | CDD:286352 |
| 4 | B6_bg3ψ | KM014056 | 7 | ABPBG3 | AIQ80447.1 | C57BL/6 | CDD:286352 |
| 5 | B6_a3 | KM014044 | 7 | ABPA3 | AIQ80459.1 | C57BL/6 | CDD:279445; CDD:238346 |
| 6 | B6_bg7 | KM014057 | 7 | ABPBG7 | AIQ80446.1 | C57BL/6 | CDD:286352 |
| 7 | B6_a7 | KM014045 | 7 | ABPA7 | AIQ80458.1 | C57BL/6 | CDD:279445; CDD:238346 |
| 8 | B6_bg10ψ | KM014058 | 7 | ABPBG10 | AIQ80445.1 | C57BL/6 | CDD:305140; CDD:286352 |
| 9 | B6_a10 | KM014046 | 7 | ABPA10 | AIQ80457 | C57BL/6 | CDD:238346 |
| 10 | B6_bg11 | KM014059 | 7 | ABPBG11 | AIQ80444.1 | C57BL/6 | CDD:286352 |
| 11 | B6_a11 | KM014047 | 7 | ABPA11_a18 | AIQ80456.1 | C57BL/6 | CDD:279445; CDD:238346 |
| 12 | B6_bg12 | KM014060 | 7 | ABPBG12 | AIQ80443.1 | C57BL/6 | CDD:286352 |
| 13 | B6_a12 | KM014048 | 7 | ABPA12 | AIQ80455.1 | C57BL/6 | CDD:279445; CDD:238346 |
| 14 | B6_bg15ψ | KM014061 | 7 | ABPBG15_bg17 | AIQ80442.1 | C57BL/6 | CDD:305140; CDD:286352 |
| 15 | B6_a15 | KM014049 | 7 | ABPA15_a17 | AIQ80454.1 | C57BL/6 | CDD:279445; CDD:238346 |
| 16 | B6_bg18 | KM014062 | 7 | ABPBG18 | AIQ80441.1 | C57BL/6 | CDD:286352 |
| 17 | B6_bg19 | KM014063 | 7 | ABPBG19 | AIQ80440.1 | C57BL/6 | CDD:286352 |
| 18 | B6_a19 | KM014050 | 7 | ABPA19 | AIQ80453.1 | C57BL/6 | CDD:279445; CDD:238346 |
| 19 | B6_bg20 | KM014064 | 7 | ABPBG20 | AIQ80439.1 | C57BL/6 | CDD:286352 |
| 20 | B6_a20 | KM014051 | 7 | ABPA20 | AIQ80452.1 | C57BL/6 | CDD:279445; CDD:238346 |
| 21 | B6_bg21 | KM014065 | 7 | ABPBG21 | AIQ80438.1 | C57BL/6 | CDD:286352 |
| 22 | B6_bg24 | KM014066 | 7 | ABPBG24 | AIQ80437.1 | C57BL/6 | CDD:286352 |
| 23 | B6_a24 | KM014052 | 7 | ABPA24 | AIQ80451.1 | C57BL/6 | CDD:279445; CDD:238346 |
| 24 | B6_bg26 | KM014067 | 7 | ABPBG26 | AIQ80436.1 | C57BL/6 | CDD:286352 |
| 25 | B6_a26ψ | KM014053 | 7 | ABPA26 | AIQ80450.1 | C57BL/6 | CDD:279445; CDD:238346 |
| 26 | B6_bg27 | KM014068 | 7 | ABPBG27 | AIQ80435.1 | C57BL/6 | CDD:286352 |
| 27 | B6_a27 | KM014054 | 7 | ABPA27 | AIQ80449.1 | C57BL/6 | CDD:279445; CDD:238346 |
| 28 | B6_a29 | KM014045 | 7 | ABPA29 | ADB46063.1 | C57BL/6 | CDD:279445; CDD:238346 |
