# Supplementary material for: Another cat and mouse game: Deciphering the evolution of the SCGB superfamily and exploring the molecular similarity of major cat allergen Fel d 1 and mouse ABP using computational approaches
Source: PLoS One. 2018 May 17;13(5):e0197618. doi: 10.1371/journal.pone.0197618 (PMC5957422; doi:10.1371/journal.pone.0197618)
Supplement: S3 Table — The similar binding site residues were highlighted as bold in Fel d 1 and ABP dimers. Also, the AB and AG dimers have similar alternate residues in the Fel d 1 dimer binding pockets. (DOCX) [file pone.0197618.s016.docx]

| Protein Name | Dimer_Pocket Number | Area (A^2^) | Volume (A^3^) | Amino acid residues with position | Other binding pocket residues |
| --- | --- | --- | --- | --- | --- |
| Fel d 1_2EJN | Subunit A_41 | 343.6 | 318.9 | Asp9, **Val10, Phe13, Leu14, Tyr21**, Val25, Leu41, Leu61, **Ile64, Phe80**, Val83, Phe84, Val87, Met112, **Ile115**, Tyr119, Asp130, Val133, **Met134** | **Cys3**, Lys29, **Glu75**, Thr76, Phe85, Ala88, Asn89, Ile125, Val128, Thr135, Ser138, Ser139, Ser140, **Cys143**, Met144 |
| Fel d 1_2EJN | Subunit B_42 | 575.4 | 682.9 | Asp9, **Val10, Phe13, Leu14, Tyr21**, Val25, **Val34**, **Asn37, Ala38**, Leu41, Leu61, **Ile64**, **Phe80**, Val83, Phe84, Val87, Ala88, Met112, **Ile115**, Tyr119, Val128, Leu129, Asp130, Gly131, Leu132, Val133, **Met134** | **Cys3**, **Glu75**, Asp82, Phe85, Asn89, Asn91, Glu92, Lys101, Leu124, Ile125, Arg127, Val128, Thr135, Ser138, Met144 |
| AB dimer | 23 | 763.7 | 684.9 | Leu6, Lys9, **Val10, Phe13, Leu14, Tyr21**, Leu25, **Val34, Asn37, Ala38**, Ile41, Ala57, Ile61, **Ile64**, **Phe80**, Ala83, Tyr84, Ile87, Phe112, **Ile115**, Phe119, Leu128, Lys129, Ser130, Pro131, Gln132, Ile133, **Met134**, Ile137 | **Cys3**, Phe28, Leu35, Ala39, Lys43, Asp46, **Glu75**, Ala78, Leu88, Glu132, Leu138, Ser140, **Cys143** |
| AG dimer | 23 | 726.9 | 724.5 | Lys9, **Val10, Phe13, Leu14, Tyr21**, Lue25, Lys33, **Val34, Asn37, Ala38**, Ile41, Ser45, Ala57, Ile61, **Ile64, Phe80**, Thr83, Tyr84, Ile87, Leu88, Arg91, Phe112, **Ile115**, Phe119, Gln124, Ile127, Ile128, Leu129, Asn130, Pro131, Gln132, Ile133 | Leu2, **Cys3**, Leu6, Phe28, Asp46, Thr65, Thr69, Cys77, Leu78, Ser79, Arg82, Gly85, Gln101, **Met134**, Leu135, Tyr138 |
