# Supplementary material for: Another cat and mouse game: Deciphering the evolution of the SCGB superfamily and exploring the molecular similarity of major cat allergen Fel d 1 and mouse ABP using computational approaches
Source: PLoS One. 2018 May 17;13(5):e0197618. doi: 10.1371/journal.pone.0197618 (PMC5957422; doi:10.1371/journal.pone.0197618)
Supplement: S4 Table — The H-bond with positions are tabulated based on members. (DOCX) [file pone.0197618.s017.docx]

| S. No | ABP accession ID | Template | Members | Structural contacts  (Fel d 1- ABP) | H-bonds | Weighted score |
| --- | --- | --- | --- | --- | --- | --- |
|  | Subunit A of Fel d 1 | | | | | |
| 1 | AGJ84407.1 (ABPAAae) | 2ejn_A | 140 | Lys29-Tyr21  Glu75-Lys29  Glu75-Lys29  Lys141-Tyr28 | 4 | -859.9 |
| 2 | NP_001257472.1 (ABPMm) | 2ejn_A | 139 | Arg8-Ile92  Arg8-Ile92  Arg8-Cys93  Lys29-Tyr44  Ser139-Lys52 | 5 | -888.4 |
| 3 | AAM08256.1 (ABPAMf) | 2ejn_A | 138 | Glu75-Arg68  Asn89-Lys9  Ser126-Thr45 | 3 | -935.2 |
| 4 | AAB97170.1 (ABPAMm) | 2ejn_A | 136 | Glu75-Arg68  Glu75-Arg68  Asn89-Lys9  Asn91-Ala5  Glu92-Asn29  Ser126-Ser45 | 6 | -914.2 |
| 5 | AAM08258.1 (ABPAMco) | 2ejn_A | 112 | Glu36-Asn37  Ala74-Arg68  Ala74-Arg68  Thr76-Arg68  Tyr81-Thr69  Asn89-Leu6  Glu123-Asn29  Ser126-Lys9  Arg127-Asn29  Arg127-Asn29  Arg127-Asn31  Ser138-Thr69 | 12 | -768.7 |
|  | Subunit B of Fel d 1 | | | | | |
| 1 | AGJ84407.1 (ABPAAae) | 2ejn_B | 172 | Met73-Cys3  Glu75-Gly1  Asp82-Lys7  Asp82-Lys7  Asn89-Lys63  Lys101-Tyr21  Arg109-Asn37  Asp130-Lys63  Asp130-Lys63 | 9 | -1061.7 |
| 2 | NP_001257472.1 (ABPMm) | 2ejn_B | 147 | Asn89-Lys86  Lys101-Asp32  Lys101-Tyr44  Glu106-Asn60  Asn103-Lys52  Arg109-Asn60  Asp130-Lys86  Asp130-Lys86 | 8 | -966.6 |
| 3 | AAM08258.1 (ABPAMco) | 2ejn_B | 121 | Glu75-Cys3  Glu75-Gly1  Asn89-Lys63  Asn91-Gln56  Lys101-Tyr21  Lys101-Lys9  Asn103-Asn29  Ala104-Asn37  Arg109-Asn37  Asn130-Lys63  Thr135-Thr69 | 11 | -787.9 |
| 4 | AAB97170.1 (ABPAMm) | 2ejn_B | 104 | Glu75-Arg68  Leu97-Lys9  Thr100-Lys9  Asn103-Asn29 | 4 | -794.1 |
| 5 | AAM08256.1 (ABPAMf) | 2ejn_B | 101 | Glu75-Gly1  Asn89-Lys63  Asn91-Gln56  Lys101-Lys9  Lys101-Tyr21  Ser139-Arg68 | 6 | -776.8 |
